# Physical Activity Recommendations Tailored by a Predictive Model for Adults With High Blood Pressure: Observational Study

**DOI:** 10.2196/78492

**Published:** 2026-01-09

**Authors:** Yuhui Yang, Manqing Chen, Weiwei Hu, Yifan Fu, Xingyan Li, Zhenli Liao, Hongman Feng, Yaling Zhao, Leilei Pei, Baibing Mi, Fangyao Chen

**Affiliations:** 1 Department of Epidemiology and Biostatistics, School of Public Health, Xi’an Jiaotong University Health Science Center, 76 Yanta Xilu Road, Xi'an, Shaanxi, 710061, China, 86 13772075793; 2 Department of Radiology, First Affiliate Hospital of Xi’an Jiaotong University, Xi’an, Shaanxi, China

**Keywords:** hypertension, physical activity pattern, machine learning, mortality, precision medicine

## Abstract

**Background:**

Whether the benefits of identical physical activity (PA) patterns for adults with high blood pressure (BP) vary according to an individual’s characteristics has not been adequately studied.

**Objective:**

This study aimed to investigate whether an individual’s characteristics modify the associations between PA patterns and mortality rate.

**Methods:**

Four PA patterns were derived from accelerometer-based data: active weekend warrior, active regular, active light PA, and baseline PA. The main outcome was all-cause mortality. A machine learning model to predict the optimal PA pattern for individual patients was trained in the UK Biobank (UKB) cohort and externally validated in the National Health and Nutrition Examination Survey cohort, which was subsequently integrated into a web-based application. The potentially optimal PA pattern within patients was identified as the one leading to the highest predicted survival probability. Multivariable Cox models were used to estimate hazard ratios and 95% CIs for all-cause mortality corresponding to the inconsistency of the current PA pattern with the predicted optimal PA pattern.

**Results:**

A total of 71,637 UKB adults and 5104 National Health and Nutrition Examination Survey individuals were enrolled. External validation demonstrated that the area under the receiver operating characteristic curve of our model for predicting mortality at 10 years of follow-up was 86.4% (95% CI 85.1%‐87.7%). The predicted optimal PA patterns in the UKB cohort were active regular PA for 26,643 (37.2%) participants, active light PA for 22,606 (31.6%) participants, and active weekend warrior for 21,749 (30.4%) participants. Stroke history, age, sex, BP class, and antihypertension medication were key factors driving heterogeneity in individuals’ optimal PA patterns. Cox regression analysis suggested that individuals in the UKB cohort whose current PA patterns were inconsistent with the predicted optimal patterns may be associated with a 28% increase in all-cause mortality risk on average (hazard ratio 1.28, 95% CI 1.20‐1.38) compared to those with consistent patterns.

**Conclusions:**

Our findings may help patients with high BP obtain individualized recommendations for PA patterns based on their specific characteristics, thereby improving their prognosis.

## Introduction

According to the latest report from the World Health Organization, the global population of adults with hypertension has more than doubled between 1990 and 2019, rising from 0.65 billion to 1.3 billion [1,2]. In 2019, high blood pressure (BP) was responsible for 10.8 million deaths worldwide, primarily due to cardiovascular diseases and chronic kidney disease [1,2]. Timely and effective interventions are therefore critical to mitigating this burden. International hypertension management guidelines universally endorse physical activity (PA) as a first-line nonpharmacological intervention for BP control [3,4]. Furthermore, PA offers distinct advantages over antihypertensive medications, including cost-effectiveness and a reduced risk of adverse effects [5,6].

As outlined by Dzau and Hodgkinson [7], patients with hypertension should receive individualized management that accounts for patients’ unique genetic and environmental factors, namely, *precision hypertension*. This perspective highlights the heterogeneity in treatment response among hypertensive individuals and underscores the need for tailored PA strategies. A major challenge in advancing precision hypertension is to move beyond the estimation of average associations and address the variability in individual responses to PA. This variability depends on factors such as personal characteristics, baseline risk profiles, and differential sensitivity to PA interventions [8]. Therefore, conducting studies on the heterogeneous associations between PA and health outcomes and identifying key factors driving this heterogeneity is essential for precision hypertension management.

PA patterns (defined by frequency, duration, and intensity of PA) and their associations with mortality have caught significant attention in recent research [9-11]. Furthermore, these studies on the heterogeneous associations between PA and mortality rely on subgroup analyses [12,13]. For example, Ji et al [13] studied the heterogeneous association between PA and mortality using prespecified gender subgroups. However, such traditional subgroup analyses require prespecification of potential subgroup variables, which limits their exploratory value [14]. Furthermore, one-variable-at-a-time analyses may produce false positives due to multiple testing and false negatives due to limited statistical power in small subgroup samples [15]. The burgeoning field of machine learning (ML) holds promise in estimating heterogeneous treatment effects, potentially offering significant support in addressing this concern [7,16]. Therefore, in our study, we adopted the S-learner framework, a metalearner for estimating individualized treatment effects using ML [17]. The ML based on metalearners can simulate the progression of hypertension and identify optimal PA patterns for managing these conditions.

We hypothesized that the associations of PA patterns with mortality would differ based on individual patient characteristics. In other words, the optimal PA pattern for patients may exhibit heterogeneity depending on individual characteristics. To test this hypothesis, we derived and externally validated a potential outcome prediction model, which was further integrated into a web-based application to identify the optimal PA pattern for an individual with hypertension. In our study, PA patterns were defined using accelerometer data from the UK Biobank (UKB) and the National Health and Nutrition Examination Survey (NHANES) cohorts.

## Methods

### Study Design and Participants

Our study included two surveys: the UKB and the NHANES. The UKB is a prospective cohort study comprising 502,629 participants enrolled between 2006 and 2010. The NHANES is a biannual survey designed to assess the health and nutritional status of the US population. This study adhered to the STROBE (Strengthening the Reporting of Observational Studies in Epidemiology) guidelines [18].

The inclusion criteria for this study were individuals with device-measured PA data and high BP. Exclusion criteria were participants with (1) poor device calibration, (2) inadequate wear time, or (3) missing values for the outcome and covariates. According to the 2024 European Society of Cardiology Guidelines for the management of elevated BP and hypertension [3], participants were classified as having high BP if they met any of the following criteria: systolic BP (SBP) ≥120 mm Hg, diastolic BP (DBP) ≥70 mm Hg, hospitalization records, or the use of antihypertensive medications. In our study, we further categorized high BP into two groups: elevated BP and hypertension. Participants with SBP between 120 and 139 mm Hg or DBP between 70 and 89 mm Hg who were not using antihypertensive medication were classified as having elevated BP; others were classified as having hypertension.

A total of 103,582 participants from the UKB and 14,631 participants from the NHANES cohort were initially considered. After applying the inclusion and exclusion criteria, 71,637 (69.2%) participants from the UKB and 5104 (34.9%) participants from NHANES were included in the final analysis.

### Exposure Ascertainment

Information on the collection of accelerometer-based PA data in the UKB and NHANES studies is provided as follows. From 2013 to 2015, UKB participants were randomly assigned to wear an Axivity AX3 accelerometer (Newcastle upon Tyne) for 7 days to measure PA and sedentary behavior. The wrist-worn accelerometers were initialized to capture data with a sampling frequency of 100 Hz and a dynamic range of ±8 g. Poor device calibration was defined as a lack of sufficient orientation changes or as having implausible acceleration values. Good wear time was defined as having at least 3 days (72 h) of data and also having data in each 1-hour period of the 24-hour cycle (scattered over multiple days). The accelerometers recorded data in milligravity units (mg). Light PA (LPA) and moderate to vigorous physical activity (MVPA) were categorized via a published ML-driven approach specifically designed for classifying a wide spectrum of activities [19].

For the NHANES, we included individuals with accelerometer-based PA data from the 2003 to 2006 cycles of NHANES. According to the design of the NHANES study, information on sedentary behavior and total PA was collected using an accelerometer (ActiGraph model 7164; ActiGraph, LLC), worn on the waist for 7 consecutive days during waking hours, except while swimming or showering. Poor device calibration was defined as more than 60 consecutive minutes with zero counts. Good wear time was defined as a recorded wear time of 10 hours or more for at least 1 day [20]. Behavior categories were defined by count per minute thresholds for adults: sedentary behavior (<100 counts per minute), LPA (100‐2020 counts per minute), and MVPA (≥2020 counts per minute) [21].

We defined four PA patterns according to the following criteria: active weekend warrior (WW; ≥150 min per week MVPA with ≥50% of total achieved in 1‐2 d) [22], active regular (≥150 min per week and not meeting MVPA WW status) [22], active LPA (<150 min per week MVPA and ≥1900 min per week LPA), and baseline PA (<150 min per week MVPA and <1900 min per week LPA). The threshold of 1900 minutes per week for an active LPA pattern was determined based on the previous study [23], and our analysis of the dose-response relationship between LPA and all-cause mortality was determined using the Cox regression models with restricted cubic splines (Multimedia Appendix 1).

### Mortality Ascertainment

The primary outcome of this study was all-cause mortality, obtained from the National Health Service Information Center (England and Wales) and the National Health Service Central Register Scotland (Scotland) for the UKB study. At the time of analysis, mortality data were available through May 31, 2024, for England and Wales, and December 31, 2023, for Scotland. For NHANES, the National Death Index was used to ascertain all-cause mortality of included samples until December 31, 2019. The follow-up period was defined as the duration between the initial PA measurement and either death or the end of follow-up, whichever occurred first. The causes of death were confirmed based on ICD-10 coding.

### Covariates

The covariates were age, sex, ethnicity, BMI, waist circumference, education, smoking status, alcohol consumption, added salt intake, sedentary time, sleep time, SBP, DBP, antihypertension medication, cancer, diabetes, myocardial infarction (MI), stroke, family cardiovascular disease (CVD), BP class, glycated hemoglobin (HbA_1c_), high-density lipoprotein cholesterol, triglyceride levels, and glucose. Ascertainment and descriptions of covariates considered for both UKB and NHANES are provided in Multimedia Appendix 2.

To minimize the time interval between covariate assessment and accelerometer-based PA measurement (typically 2013‐2015) in the UKB, we prioritized data from instance 1 (the repeat assessment in 2012‐2013) when both instance 0 (the baseline assessment in 2006‐2010) and instance 1 were available [22]. We assessed the reliability of covariates in participants with repeated measurements by the intraclass correlation coefficient [24] for continuous variables and Cohen κ [25] for categorical variables. The analysis indicated moderate to high reliability of the covariates; detailed results are provided in Multimedia Appendix 3. In contrast, the PA and covariates in the external validation set, NHANES, were measured during the same period, thus avoiding the aforementioned issues present in UKB.

### Model Development and Estimation of Heterogeneous Associations

We randomly divided the UKB data into an 80% training set and a 20% internal validation set, with the NHANES dataset used for external validation. Predictors were selected using Cox regression with the least absolute shrinkage and selection operator (LASSO) penalization [26], where the optimal shrinkage parameter lambda was determined via 10-fold cross-validation [27]. The original predictive model was constructed using a multivariable Cox model that incorporated all second-order interactions between PA pattern and the selected predictors. Then, we applied a stepwise backward elimination method to refine the model, removing variables and interaction terms that did not significantly contribute to the model [28]. Model performance was evaluated in the internal validation set and the NHANES validation set and visualized using the calibration curves (with Brier score) and receiver operating characteristic (ROC) analysis (with the area under the ROC curve [AUC]) [29,30].

Our S-learner algorithm for estimating heterogeneous associations between PA and overall survival follows a 2-step process. First, it uses the trained prediction model to estimate the conditional expectations of survival time for each of the 4 PA patterns separately. Second, it computes the differences between these estimates to capture the heterogeneous associations.

On the basis of the S-learner framework, the trained prediction model was capable of handling potential variations in covariates, enabling the prediction of each patient’s potential survival probabilities under the 4 PA patterns. Then, the best potential PA pattern for a certain patient was identified as the one leading to the highest predicted survival probability. To operationalize these findings, we developed an R Shiny web application that enables clinicians to simulate personalized survival curves for all 4 PA patterns based on individual baseline characteristics.

### Patient Characteristics Associated With Predicted Individualized PA Patterns

All individuals in the UKB and the NHANES cohorts were stratified by PA patterns that might individually optimize overall survival. This stratification was used to identify the profiles of individuals who could benefit most from 1 of the 4 PA patterns presented, according to the constructed prediction model.

To facilitate rapid decision-making and clearly illustrate patient characteristics associated with predicted individualized PA patterns, a model of conditional inference tree (CIT) was established [31]. Specifically, we used the potentially optimal PA patterns predicted as the outcome and all covariates as predictors to build the CIT model.

### Statistical Analysis

We introduced an indicator variable, referred to as an “inconsistent PA pattern,” to distinguish between individuals whose actual and predicted optimal PA patterns were inconsistent and those whose patterns were consistent. Multivariable Cox models were used to estimate hazard ratios (HRs) and 95% CIs for all-cause mortality corresponding to the inconsistency of the current PA pattern with the predicted optimal PA pattern. The analyses were adjusted for covariates selected by LASSO.

As further stratification of participants with inconsistent PA patterns provides more clinically meaningful information, we subdivided the inconsistent group into two categories based on the predicted optimal intensity: (1) inconsistent MVPA pattern—participants whose predicted optimal PA pattern required more MVPA but whose actual activity was lower than predicted and (2) inconsistent LPA pattern—participants whose predicted optimal PA pattern required LPA primarily but whose actual activity (including active regular, active WW, and baseline PA patterns) differed. The original binary variable “inconsistent PA pattern” was thus updated to a category variable with three levels: consistent, inconsistent MVPA pattern, and inconsistent LPA pattern. We then used “consistent” as a reference class for the aforementioned multivariable Cox model analyses. In addition, we conducted subgroup analysis in the actually observed nonbaseline group.

### Sensitivity Analyses

To test the generalizability of the aforementioned analyses, we performed 4 prespecified sensitivity analyses. First, we repeated the statistical analyses using the NHANES dataset. Second, we replaced the all-cause mortality outcome with CVD-specific mortality and cancer-specific mortality outcomes. Third, to minimize the potential confounding effects of the COVID-19 pandemic, we restricted follow-up to the period before December 31, 2019. This prepandemic dataset was used as an additional validation cohort to assess the robustness of the model’s performance and to repeat the statistical analyses. Finally, the statistical analyses were repeated using the internal validation set.

All analyses were conducted using R version 4.4.1 (R Foundation), with statistical significance set at *P<.05*.

### Ethical Considerations

This study involved secondary analysis of data from the UKB and the NHANES, whose original data collection protocols were conducted in accordance with the Declaration of Helsinki. UKB was approved by the North West Multi-centre Research Ethics Committee (reference 21/NW/0157), and all participants provided written informed consent for health-related research, including secondary analyses. The NHANES study received ethical approval from the National Center for Health Statistics Ethics Review Board. The National Center for Health Statistics Ethics Review Board approved Protocol 98‐12 for the 2003‐2004 cycle and Protocol 2005‐06 for the 2005‐2006 cycle. All participants provided written informed consent. All data were anonymized or deidentified.

## Results

### Overview

**Figure 1.**
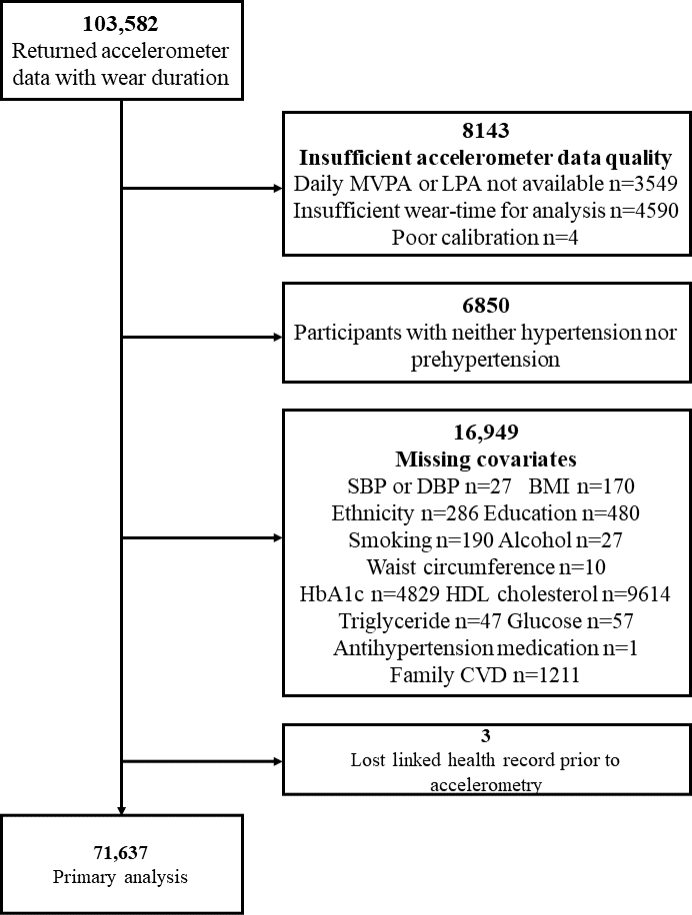
Study flow for UK Biobank. CVD: cardiovascular disease; DBP: diastolic blood pressure; HbA_1c_: glycated hemoglobin; HDL: high-density lipoprotein; LPA: light physical activity; MVPA: moderate to vigorous physical activity; SBP: systolic blood pressure.

For the UKB cohort, a total of 71,637 adults were enrolled and analyzed (Figure 1), with 4207 deaths confirmed during a median follow-up period of 9.5 years. For the NHANES cohort, we included 5104 individuals who underwent device-based activity measurements between the 2003 and 2006 cycles (Multimedia Appendix 4), with 1279 deaths confirmed during a median follow-up period of 14.3 years. The median follow-up time for survivors and nonsurvivors in both the UKB and the NHANES datasets is shown in Multimedia Appendix 5. The baseline characteristics of participants, stratified by 4 actually *observed* PA patterns, are summarized in Multimedia Appendix 6 for the UKB cohort and the NHANES cohort. At baseline in the UKB, 12,577 (17.6%) participants were classified as baseline PA, 12,782 (17.8%) participants were classified as active LPA, 15,141 (21.1%) participants were classified as active regular, and 31,137 (43.5%) participants were classified as active WW.

### A Prediction Model to Identify the Best Potential PA Patterns

The detailed results from the LASSO-penalized Cox model for covariate selection are presented in Multimedia Appendix 7. In brief, the selected covariates included PA patterns, age, sex, sedentary time, smoking status, antihypertension medication, cancer, diabetes, MI, stroke, BP class, waist circumference, glucose, and HbA_1c_. In the UKB cohort, the associations between covariates selected by LASSO and all-cause mortality were examined using univariate Cox models (Multimedia Appendix 8): PA patterns were found to be associated with overall survival, suggesting that adults with active LPA (HR 0.56, 95% CI 0.51‐0.62), active regular (HR 0.45, 95% CI 0.41‐0.50), or active WW (HR 0.47, 95% CI 0.43‐0.51) were at a lower risk of death than those with the baseline PA pattern.

**Figure 2.**
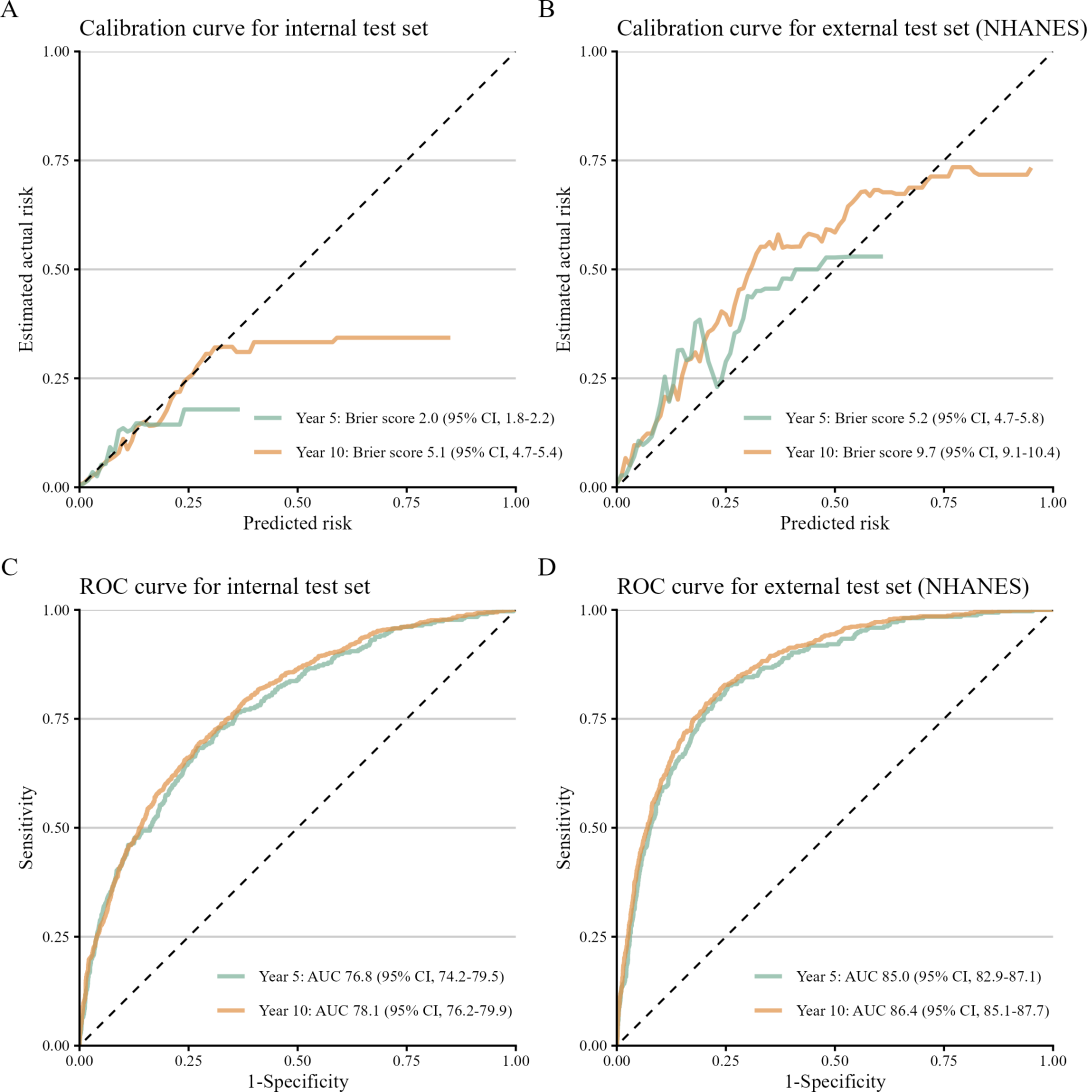
Performance of the model in predicting all-cause mortality. The calibration and ROC curves for internal (n=14,328) and external test data set (n=5104). AUC: area under the receiver operating characteristic curve; NHANES: National Health and Nutrition Examination Survey; ROC: receiver operating characteristic.

The original predictive model incorporated all second-order interactions of PA patterns with each selected covariate. Following stepwise backward elimination, the main effect of glucose and the interactions of PA patterns with waist circumference, smoking status, and glucose were removed from the model. The terms and coefficients of the final model are detailed in Multimedia Appendix 9. The model showed well-calibrated predictions in the internal test set, with Brier scores of 2.0% (95% CI 1.8%‐2.2%) for the 5-year and 5.1% (95% CI 4.7%‐5.4%) for the 10-year prediction (Figure 2A). Calibration remained acceptable in the external NHANES set, although it was slightly decreased, with Brier scores of 5.2% (95% CI 4.7%‐5.8%) for the 5-year and 9.7% (95% CI 9.1%‐10.4%) for the 10-year prediction (Figure 2B). The model also demonstrated good discriminative ability. In the internal set, the AUC was 76.8 % (95% CI 74.2%‐79.5%) for the 5-year and 78.1% (95% CI 76.2%‐79.9%) for the 10-year prediction (Figure 2C). This performance was further improved in the external validation set, with AUCs of 85.0% (95% CI 82.9%‐87.1%) and 86.4% (95% CI 85.1%‐87.7%) for the 5- and 10-year prediction, respectively (Figure 2D).

The prediction model identifying the best PA pattern has been integrated into an online application, accessible free of charge (Multimedia Appendix 10). To ensure broader accessibility of the application, we have provided both English and Chinese versions (Multimedia Appendix 10). Users need to enter the required clinical characteristics (including age, sex, sedentary time, smoking status, antihypertension medication, cancer, diabetes, MI, stroke, BP class, waist circumference, and HbA_1c_) into the website. The tool then calculates the predicted 5-year and 10-year survival probabilities corresponding to the 4 PA patterns. After these probabilities are generated, the application identifies the pattern with the highest predicted survival probability. This information can support clinicians and patients in selecting the most appropriate personalized PA pattern.

### Patient Characteristics Associated With Predicted Optimal PA Patterns

Our predictive model indicated that the optimal PA pattern varied among adults with high BP. Specifically, 26,643 (37.2%) participants were predicted to benefit most from the active regular pattern, 22,606 (31.6%) participants from the active LPA pattern, 21,749 (30.4%) participants from the active WW pattern, and 639 (0.9%) participants from the baseline PA pattern. Table 1 presents the characteristics of participants according to their *predicted* optimal PA patterns and compares these with the patterns they actually followed in the UKB cohort. In addition, we applied the same predictive approach to NHANES participants and compared *predicted* versus *observed* PA patterns, as shown in Multimedia Appendix 11.

**Table 1.** Baseline characteristics of participants stratified by the predicted optimal physical activity (PA) pattern.

Variables and levels		Baseline PA (n=639)	Active LPA^a^ (n=22,606)	Active regular (n=26,643)	Active WW^b^ (n=21,749)	*P* value
Pattern actually observed, n (%)						<.001
Baseline PA		191 (29.9)	3118 (13.8)	5238 (19.7)	4030 (18.5)	
Active LPA		118 (18.5)	4532 (20.0)	4734 (17.8)	3398 (15.6)	
Active regular		105 (16.4)	5169 (22.9)	5467 (20.5)	4400 (20.2)	
Active WW		225 (35.2)	9787 (43.3)	11,204 (42.1)	9921 (45.6)	
Age (y), median (IQR)		67.9 (63.7-71.3)	56.8 (51.7-62.4)	62.8 (56.9-67.3)	69.8 (66.8-72.5)	<.001
Sex, n (%)						<.001
Male		447 (70)	4407 (19.5)	12,069 (45.3)	15,948 (73.3)	
Female		192 (30)	18,199 (80.5)	14,574 (54.7)	5801 (26.7)	
WC^c^ (cm), median (IQR)		96.0 (87.0-104.2)	82.0 (75.0-91.0)	90.0 (81.0-99.0)	93.0 (85.0-101.0)	<.001
Smoking, n (%)						<.001
Never		283 (44.3)	14,066 (62.2)	15,541 (58.3)	10,943 (50.3)	
Previous		300 (46.9)	6979 (30.9)	9206 (34.6)	9639 (44.3)	
Current		56 (8.8)	1561 (6.9)	1896 (7.1)	1167 (5.4)	
Antihypertension medication, n (%)						<.001
No		217 (34)	19,694 (87.1)	23,541 (88.4)	14,643 (67.3)	
Yes		422 (66)	2912 (12.9)	3102 (11.6)	7106 (32.7)	
Blood pressure class, n (%)						<.001
Elevated		54 (8.5)	19,513 (86.3)	3405 (12.8)	9907 (45.6)	
Hypertension		585 (91.5)	3093 (13.7)	23,238 (87.2)	11,842 (54.4)	
MI^d^, n (%)						<.001
No		556 (87)	22,595 (100)	26,097 (98)	20,705 (95.2)	
Yes		83 (13)	11 (0)	546 (2)	1044 (4.8)	
Stroke, n (%)						<.001
No		0 (0)	22,527 (99.7)	26,585 (99.8)	21,409 (98.4)	
Yes		639 (100)	79 (0.3)	58 (0.2)	340 (1.6)	
Diabetes, n (%)						<.001
No		573 (89.7)	21,610 (95.6)	26,454 (99.3)	19,405 (89.2)	
Yes		66 (10.3)	996 (4.4)	189 (0.7)	2344 (10.8)	
Cancer, n (%)						<.001
No		534 (83.6)	22,291 (98.6)	22,158 (83.2)	20,519 (94.3)	
Yes		105 (16.4)	315 (1.4)	4485 (16.8)	1230 (5.7)	
Glucose (mmol/L), median (IQR)		5.0 (4.7-5.5)	4.8 (4.5-5.2)	5.0 (4.6-5.3)	5.0 (4.6-5.4)	<.001
HbA_1c_^e^ (mmol/mol), median (IQR)		36.9 (34.5-39.8)	33.8 (31.5-36.1)	35.5 (33.2-37.9)	35.5 (33.1-38.1)	<.001

a LPA: light physical activity.

b WW: weekend warrior.

c WC: waist circumference.

d MI: myocardial infarction.

e HbA_1c_: glycated hemoglobin.

**Figure 3.**
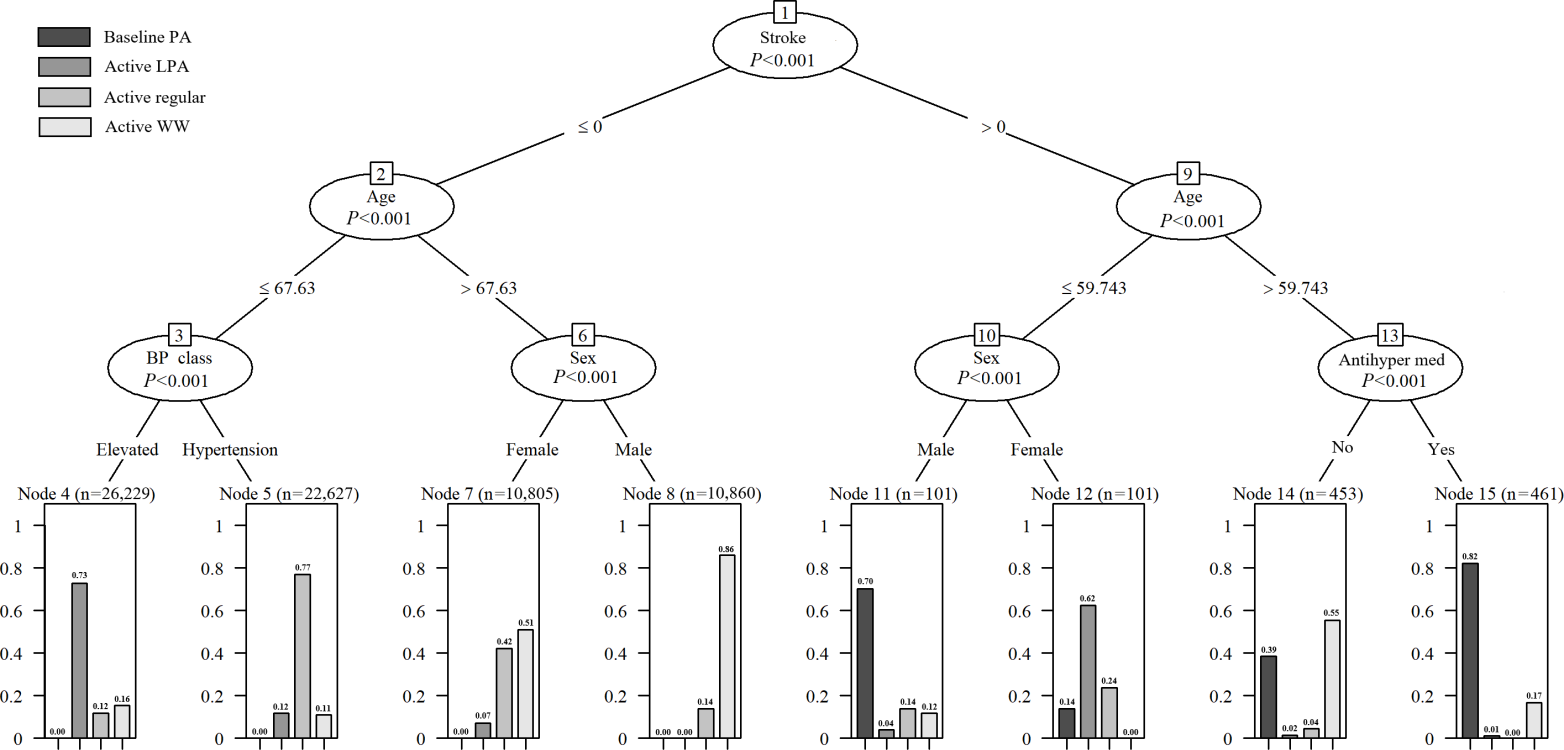
The derived conditional inference tree. The models were constructed by the optimal PA pattern predicted (outcome) and all covariates selected (predictors). Antihyper med: antihypertension medication; BP: blood pressure; LPA: light physical activity; WW: weekend warrior.

The results of the CIT model are shown in Figure 3. Briefly, the model is rooted by stroke, with subsequent branching by age, BP class, sex, and antihypertension medication (all *P*<.001). Adults with stroke are more likely to have baseline PA or active LPA as optimal patterns. In contrast, for those without stroke, younger adults (≤68 y) tend to benefit more from active regular and active LPA, whereas older men (>68 y) are predicted to gain more from the active WW pattern. In summary, stroke, age, sex, BP class, and antihypertension medication are key factors driving heterogeneity in individuals’ optimal PA patterns. Notably, the baseline PA pattern denotes a lower level of PA rather than complete physical inactivity or sedentary behavior.

### The Associations Between Inconsistent PA Patterns and Mortality

**Figure 4.**
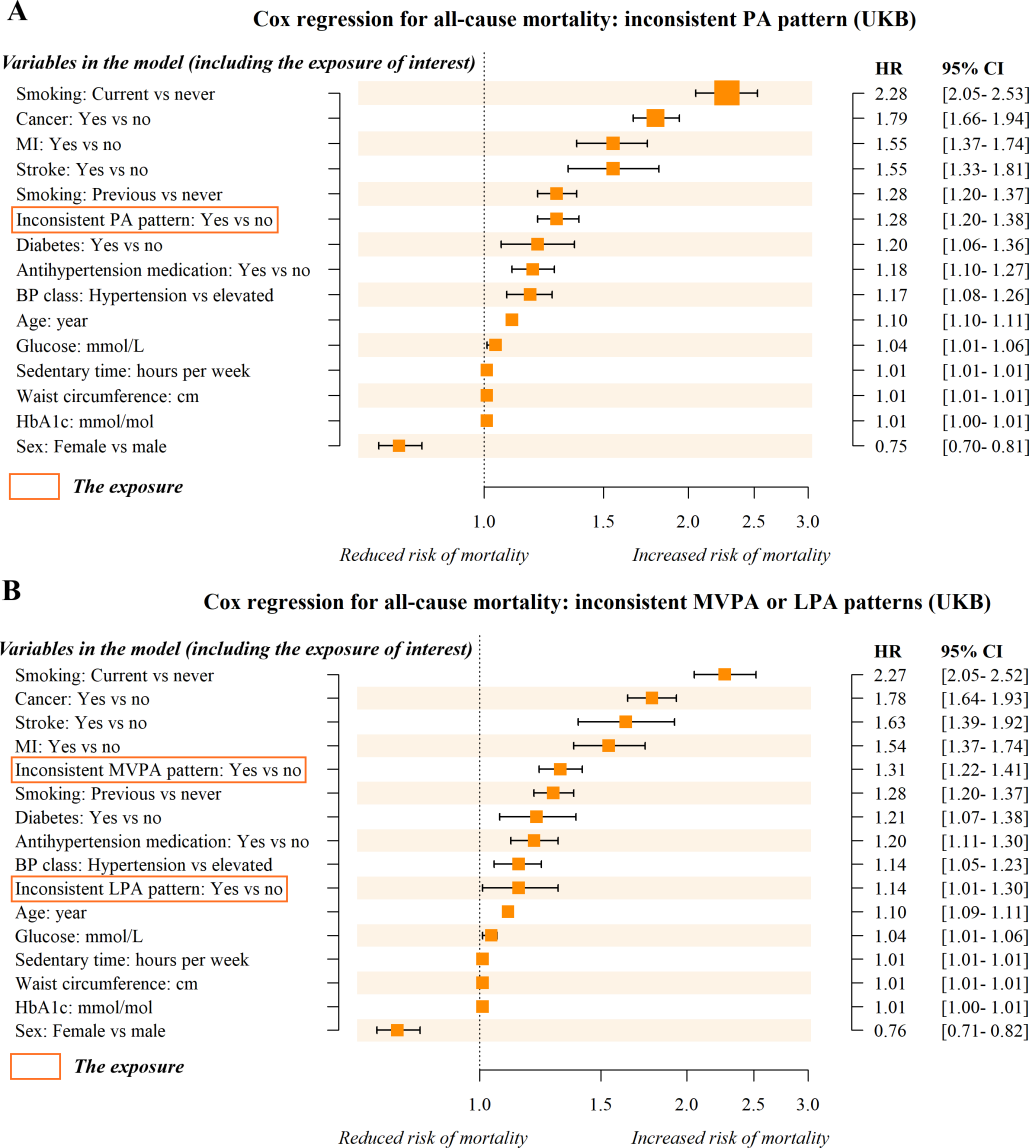
Forest plot of hazard ratios (HRs) from the multivariate Cox regression model. Outcome: all-cause mortality. Exposure: inconsistent PA patterns (distinguishing between individuals with consistent [actual vs predicted optimal PA patterns] and inconsistent PA patterns). In Figure 4A, the exposure is a binary variable (consistent vs inconsistent); in Figure 4B, it is a categorical variable with 3 levels (consistent [reference group], inconsistent MVPA pattern, and inconsistent LPA pattern). The Cox regressions were analyzed and sorted by HR. BP: blood pressure; HbA_1c_: glycated hemoglobin; LPA: light physical activity; MVPA: moderate to vigorous physical activity; PA: physical activity; UKB: UK Biobank.

**Figure 5.**
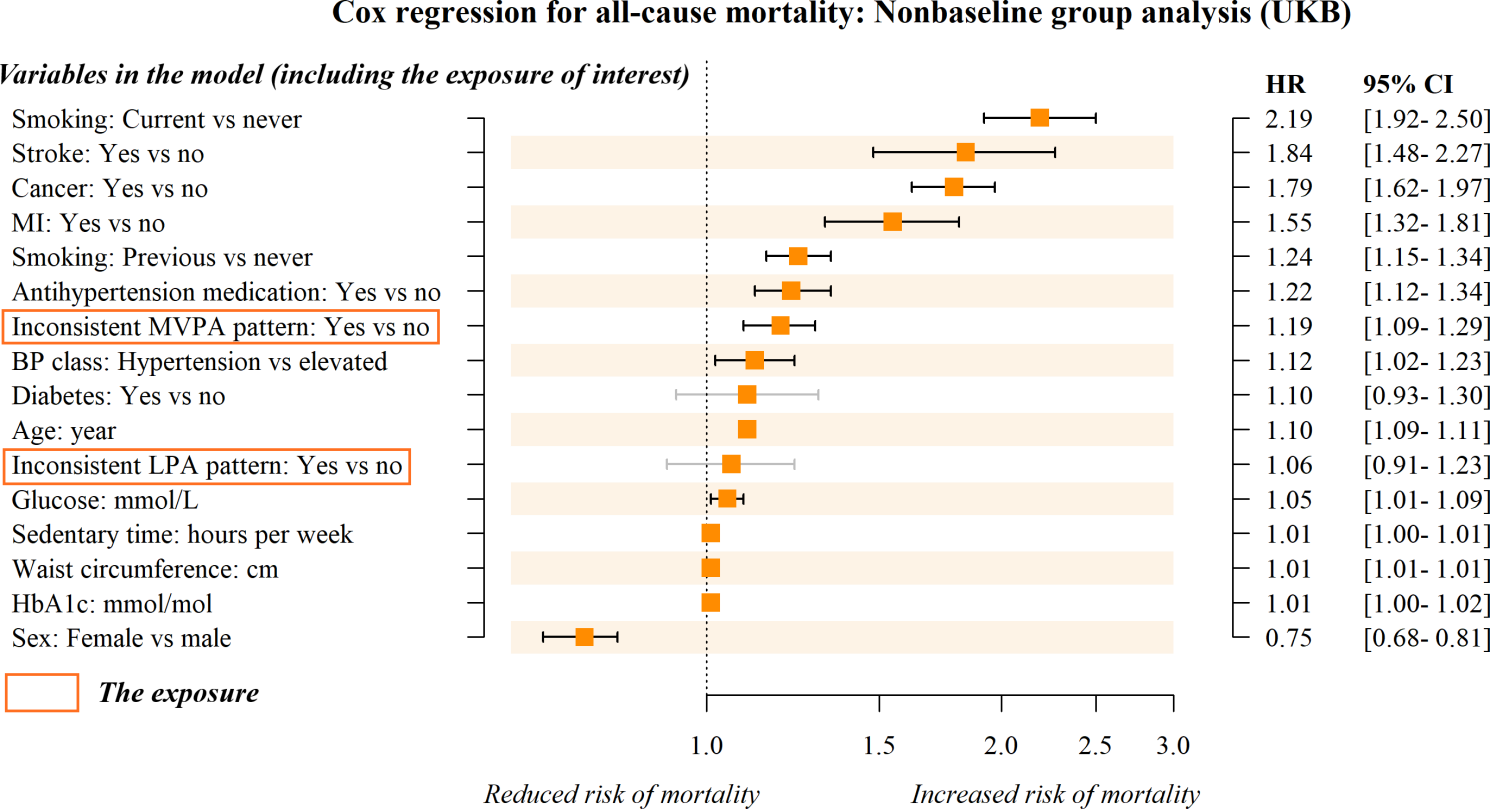
Forest plot of hazard ratios (HRs) from the multivariate Cox regression model in the nonbaseline subgroup. Outcome: all-cause mortality. Exposure: inconsistent PA patterns (distinguishing between individuals with consistent [actual vs predicted optimal PA patterns] and inconsistent PA patterns) with 3 levels (consistent [reference group], inconsistent MVPA pattern, and inconsistent LPA pattern). The nonbaseline group comprises participants whose actually observed PA pattern was not the “baseline PA” pattern. The Cox regressions were analyzed and sorted by HR. BP: blood pressure; HbA_1c_: glycated hemoglobin; LPA: light physical activity; MVPA: moderate to vigorous physical activity; PA: physical activity; UKB: UK Biobank.

The PA patterns observed in 71.9% of participants in the UKB cohort and 78.9% of participants in the NHANES cohort were inconsistent with the optimal patterns predicted by our model. Compared to individuals whose actual and predicted optimal PA patterns were consistent, an inconsistent PA pattern was associated with a 28% increase in all-cause mortality risk on average (HR 1.28, 95% CI 1.20‐1.38; Figure 4A) in the UKB cohort. Specifically, an inconsistent MVPA pattern was associated with a 31% increased risk (HR 1.31, 95% CI 1.22‐1.41; Figure 4B), whereas an inconsistent LPA pattern was associated with a 14% increased risk (HR 1.14, 95% CI 1.01‐1.30; Figure 4B). In subgroup analysis, we found no statistically significant association between the inconsistent LPA pattern and all-cause mortality (HR 1.06, 95% CI 0.91‐1.23; Figure 5).

### Sensitivity Analyses

The results of the sensitivity analyses are demonstrated in Multimedia Appendix 12. The results of the first sensitivity analysis using the NHANES data showed that an inconsistent PA pattern was associated with a 26% increase in all-cause mortality risk (HR 1.26, 95% CI 1.06‐1.51), compared to individuals with consistent actual and predicted optimal PA patterns. The analyses of the associations between inconsistent MVPA or LPA patterns and mortality, as well as subgroup analyses, also yielded results similar to those in the UKB cohort. The results of the second sensitivity analysis using cause-specific mortality outcomes indicated that an inconsistent PA pattern was associated with an 18% increase in cancer mortality risk (HR 1.18, 95% CI 1.07‐1.30) and a 33% increase in CVD mortality risk (HR 1.33, 95% CI 1.18‐1.49). Subgroup analyses revealed that the inconsistent LPA pattern was not statistically significantly associated with either cancer or CVD mortality in the UKB cohort. In the third sensitivity analysis, we used the prepandemic dataset for external validation. The results showed that the Brier score of our model for predicting 5-year mortality was 2.0% (95% CI 1.8%‐2.3%), and the AUC was 78.1% (95% CI 75.3%‐80.8%). In addition, both the overall and subgroup analyses indicated no statistically significant associations between the inconsistent LPA pattern and all-cause mortality. The results of the fourth sensitivity analysis were consistent with those of the primary analysis.

## Discussion

### Principal Findings

On the basis of the analysis of 2 temporally and geographically distinct cohorts, we found that patients’ individual characteristics modified the benefit of PA patterns on all-cause mortality. Our findings in recognition of the best potential PA pattern revealed that up to 67.6% of patients with high BP were advised to engage in over 150 minutes of MVPA per week (37.2% for active regular and 30.4% for active WW). However, we also found that only 31.8% of them were actually with the predicted optimal PA pattern. In other words, according to the prediction model, a significant portion of the participants may not follow the most suitable PA pattern based on their underlying personal characteristics. Moreover, we found that the risk of death was significantly lower when current PA patterns were consistent with the predicted optimal patterns. This suggests the urgent need to implement standardized, individualized, and evidence-based PA pattern recommendations for patients with hypertension to enhance their prognosis.

Our subgroup analysis of the nonbaseline participants revealed no statistically significant associations between the inconsistent LPA pattern and all-cause mortality. As the subgroup with an inconsistent LPA pattern among nonbaseline participants was predominantly engaged in MVPA, this null finding suggests that increasing PA intensity and volume beyond the predicted levels may not offer additional benefits. Therefore, identifying such subgroups may be important, as simple goals could increase patients’ confidence [32] and adherence [33] to PA engagement. However, while our study strongly supports the importance of meeting the minimum PA intensity threshold, evidence on the potential harm of excessive exercise beyond the predicted level remains weak. This warrants further studies with stronger causal evidence, such as randomized controlled trials [34,35].

Hypertension frequently coexists with cardiovascular disease, obesity, diabetes, or cognitive decline, particularly among older adults [36-39]. These comorbidities complicate PA management in hypertensive patients due to two key challenges: (1) older people with comorbidities tend to have limited physical ability to engage in MVPA [40] and (2) VPA may attenuate PA’s cardiovascular benefits by inducing adverse cardiac remodeling in vulnerable individuals [41-43]. Consequently, PA regimens should be tailored to individual physical conditions, and our model provides insights to guide this personalization. For instance, the identified interaction terms reveal that the associations between specific PA patterns and mortality differ by comorbidity. Specifically, for patients with MI who are engaged in cardiac rehabilitation, regular MVPA may offer greater benefits [44]. Hypertensive individuals with diabetes may benefit more from light-intensity PA, as excessive exercise could lead to hypoglycemia [45]. Additionally, participants with a stroke comorbidity are more likely to benefit from a lower level of PA due to the physical limitations and health risks associated with stroke recovery. Light-intensity PA offers a safer, more sustainable option, promoting cardiovascular health and functional recovery without overwhelming the individual [46,47]. However, for high-comorbidity groups, the model-predicted optimal pattern may serve as a prognostic marker rather than a prescriptive target for exercise limitation due to potential unmeasured confounders.

Advances in artificial intelligence have the potential to make personalized health advice more accessible and affordable for a broader range of individuals [48,49]. The model derived and online application proposed in this study could simulate various prognostic scenarios under different PA patterns for adults with hypertension and help patients choose the individualized PA pattern. Importantly, our algorithm can be integrated into current practices that rely on in-person assessments and regular follow-ups, as a support but not as a replacement for the physicians’ professionalism and experience [50].

### Study Limitations

This study also has several limitations. First, PA patterns were assessed at baseline using a short-term (7 d) estimate, whereas the median follow-up was 9.5 years for UKB and 14.3 years for NHANES. This may not accurately reflect typical PA patterns over time. Additionally, our predictive model assumes a static PA pattern, which limits its clinical applicability. Second, although we prioritized covariate data from the repeat assessment (2012‐2013) to align with the accelerometer measurement period (2013‐2015), most of the covariate data were sourced from the baseline assessment (2006‐2010). This introduces the risk of measurement error due to temporal changes in lifestyle or health status, thereby compromising the tool’s real-world predictive accuracy. Third, the ML model we developed was based on the observation survey rather than a randomized controlled trial, so the capacity of our results to elucidate causal relationships is limited. Fourth, the observed association between the “inconsistent PA pattern” and higher mortality risk may be influenced by residual confounding from unmeasured health-seeking behaviors. While our sensitivity analysis results have enhanced the reliability of our conclusions, the unmeasured confounders cannot be overlooked. Fifth, the majority of participants were White, which limits the generalizability of the model and means its predictions may have limited applicability to non-White populations. Sixth, the prediction model was trained and validated using objective accelerometer data; however, the use of self-reported sedentary time as input in the web application may result in inaccurate survival predictions [51]. In addition, the reliance on objective sedentary time and the need for recent blood biochemical marker data may limit the tool’s applicability to the general public and primary prevention. Finally, as the optimal PA pattern driven by small increases in survival probability may lack clinical significance, establishing an appropriate threshold should be an important focus for future research. In addition, the threshold selection of the “active LPA pattern” merits further inquiry to verify its biological and clinical significance.

### Conclusions

Our study found that the optimal PA pattern was heterogeneous based on individuals’ underlying characteristics, whereas only a few participants actually followed the most suitable PA pattern as we predicted. The algorithm presented herein could assist in assigning patients with high BP to the individualized PA pattern based on their specific characteristics, which can be easily accessed through an online application.

## Supplementary material

10.2196/78492 Multimedia Appendix 1 The analysis of the dose-response relationship between light physical activity and all-cause mortality.

10.2196/78492 Multimedia Appendix 2 Covariates ascertainment and descriptions.

10.2196/78492 Multimedia Appendix 3 The reliability measurements of covariates.

10.2196/78492 Multimedia Appendix 4 Study flow for National Health and Nutrition Examination Survey.

10.2196/78492 Multimedia Appendix 5 Time to censoring and time to death for the UK Biobank and National Health and Nutrition Examination Survey sets.

10.2196/78492 Multimedia Appendix 6 Baseline characteristics of participants.

10.2196/78492 Multimedia Appendix 7 The detailed results obtained using the least absolute shrinkage and selection operator penalized Cox model for covariate selection.

10.2196/78492 Multimedia Appendix 8 The associations between covariates selected and all-cause mortality by univariate Cox models in the UK Biobank cohort.

10.2196/78492 Multimedia Appendix 9 Coefficients and SEs of the Cox prediction model.

10.2196/78492 Multimedia Appendix 10 A snapshot from the web application.

10.2196/78492 Multimedia Appendix 11 A baseline characteristic of participants stratified by the predicted optimal physical activity pattern in the National Health and Nutrition Examination Survey cohort.

10.2196/78492 Multimedia Appendix 12 The results of the sensitivity analyses.

## Abbreviations

AUC: area under the receiver operating characteristic curve
BP: blood pressure
CIT: conditional inference tree
CVD: cardiovascular disease
DBP: diastolic blood pressure
HbA_1c_: glycated hemoglobin
HR: hazard ratio
LASSO: least absolute shrinkage and selection operator
LPA: light physical activity
MI: myocardial infarction
ML: machine learning
MVPA: moderate to vigorous physical activity
NHANES: National Health and Nutrition Examination Survey
PA: physical activity
ROC: receiver operating characteristic
SBP: systolic blood pressure
STROBE: Strengthening the Reporting of Observational Studies in Epidemiology
UKB: UK Biobank
WW: weekend warrior
