# Supplementary material for: Physical Activity Recommendations Tailored by a Predictive Model for Adults With High Blood Pressure: Observational Study
Source: J Med Internet Res. 2026 Jan 9;28:e78492. doi: 10.2196/78492 (PMC12788716; doi:10.2196/78492)
Supplement: Multimedia Appendix 1 [file jmir-v28-e78492-s001.docx]

**Multimedia Appendix 2.** The analysis of the dose-response relationship between LPA and all-cause mortality


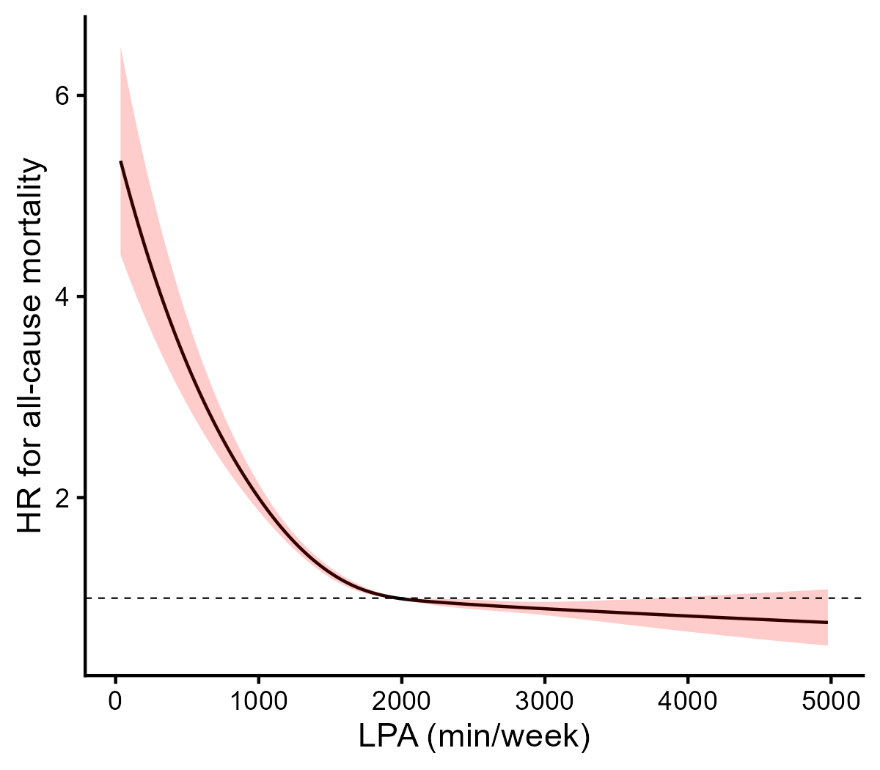


Figure 1: Dose-response association of LPA with all-cause mortality by the Cox regression models with restricted cubic splines
