# Supplementary material for: Physical Activity Recommendations Tailored by a Predictive Model for Adults With High Blood Pressure: Observational Study
Source: J Med Internet Res. 2026 Jan 9;28:e78492. doi: 10.2196/78492 (PMC12788716; doi:10.2196/78492)
Supplement: Multimedia Appendix 2 [file jmir-v28-e78492-s002.docx]

**Multimedia Appendix 3.** Covariates ascertainment and descriptions

Table 1: Covariates ascertainment and descriptions for UKB.

| **Variables** | **UK Biobank Field ID(s)** | **Instance(s)** | **Description** | **Coding** | **Notes*** |
| --- | --- | --- | --- | --- | --- |
| Age | 21022 | 0 | Age | Continuous age  in years | Age calculated at the end of accelerometer wear. |
| Sex | 31 | 0 | Sex | 1) Male  2) Female |  |
| Ethnicity | 21000 | 0 | Ethnic background | 1) White  2) Nonwhite | 1) White: British, Any other white background, Irish, White; 2) Nonwhite: White and Asian, Caribbean, Chinese, Pakistani, White and Black African, Other ethnic group, Any other mixed background, African, White and Black Caribbean, Indian, Any other Black background, Any other Asian background, Bangladeshi, Mixed, Asian or Asian British, Black or Black British. |
| BMI (Kg/m^2^) | 21001 | 0, 1† | Body mass index (BMI) | Continuous in  kg/m2 |  |
| Waist circumference | 48 | 0, 1† | Waist circumference | Continuous in  cm |  |
| Education | 6138 | 0, 1† | Qualifications | 1) School leaver  2) Further education  3) Higher education | 1) School leaver: O levels/GCSEs or equivalent, CSEs or equivalent, None of above; 2) Further education: A levels/AS levels or equivalent, NVQ or HND or HNC or equivalent, Other professional qualifications: nursing, teaching; 3) Higher education: College or University degree. |
| Smoking | 20116 | 0, 1† | Smoking status | 1) Never  2) Previous  3) Current |  |
| Alcohol | 1558 | 0, 1† | Alcohol intake frequency | 1) <3 times/week  2) 3+ times/week | 1) <3 times/week: Special occasions only, One to three times a month, Once or twice a week; 2) 3+ times/week: Three or four times a week, Daily or almost daily. |
| Added salt | 1478 | 0, 1† | Salt added to food | 1) Never/rarely  2) Sometimes  3) Usually  4) Always |  |
| Sedentary time | 40047 | 2013-2016 | Sedentary - Overall average | Continuous in hours/week | Sedentary time = Sedentary - Overall average * Wear duration overall |
|  | 90051 | 2013-2016 | Wear duration overall |  |  |
| Sleep time | 40046 | 2013-2016 | Sleep - Overall average | Continuous in hours/week | Sleep time = Sleep - Overall average * Wear duration overall |
|  | 90051 | 2013-2016 | Wear duration overall |  |  |
| SBP | 93 | 0, 1† | Systolic blood pressure, manual reading | Continuous in mmHg | SBP = (Systolic blood pressure, manual reading + Systolic blood pressure, automated reading)/2; If there are missing values in manual reading or automated reading, use the non-missing values. |
|  | 4080 | 0, 1† | Systolic blood pressure, automated reading |  |  |
| DBP | 94 | 0, 1† | Diastolic blood pressure, manual reading | Continuous in mmHg | DBP = (Diastolic blood pressure, manual reading + Diastolic blood pressure, automated reading)/2; If there are missing values in manual reading or automated reading, use the non-missing values. |
|  | 4079 | 0, 1† | Diastolic blood pressure, automated reading |  |  |
| Antihypertension medication | 6177 | 0, 1† | Medication for cholesterol, blood pressure or diabetes | 1) No  2) Yes |  |
|  | 6153 | 0, 1† | Medication for cholesterol, blood pressure, diabetes, or take exogenous hormones |  |  |
| Blood pressure class | 41202 | Hospital data | Diagnoses - main ICD10 | 1) elevated  2) hypertension | ICD10: I10, I11, I12, I13, I15. Date of hypertension occurrences should be before the time of PA measurement. Participants with SBP between 120 and 139 mmHg or DBP between 70 and 89 mmHg who were not using antihypertensive medication were classified as elevated; others were considered hypertensive. |
|  | 41204 | Hospital data | Diagnoses – secondary ICD10 |  |  |
| Diabetes | 130706  130708  130710  130712  130714 | The ‘first occurrence’ of any code mapped to 3-character ICD-10 | Date E11-E14 first reported | 1) No  2) Yes | Date of diabetes occurrences should be before the time of PA measurement. |
|  | 2986 | 0, 1† | Started insulin within one year diagnosis of diabetes |  | / |
|  | 2443 | 0, 1† | Diabetes diagnosed by doctor |  | / |
|  | 6177 | 0, 1† | Medication for cholesterol, blood pressure or diabetes |  | / |
|  | 6153 | 0, 1† | Medication for cholesterol, blood pressure, diabetes, or take exogenous hormones |  | / |
| Cancer | 41202 | Hospital data | Diagnoses - main ICD10 | 1) No  2) Yes | ICD10: C00-C97; Date of cancer occurrences should before time of PA measurement. |
|  | 41204 | Hospital data | Diagnoses – secondary ICD10 |  |  |
| MI | 131298  131300 | The ‘first occurrence’ of any code mapped to 3-character ICD-10 | Date I21-I22 first reported | 1) No  2) Yes |  |
| Stroke | 131360  131362  131364  131366  131368 | The ‘first occurrence’ of any code mapped to 3-character ICD-10 | Date I60-I64 first reported | 1) No  2) Yes |  |
| Family CVD | 20107 | 0, 1† | Illnesses of father  Family history | 1) No  2) Yes | Father, mother, or siblings with stroke or heart disease |
|  | 20110 | 0, 1† | Illnesses of mother  Family history |  |  |
|  | 20111 | 0, 1† | Illnesses of siblings  Family history |  |  |
| HbA1c | 30750 | 0, 1† | Glycated haemoglobin (HbA1c) | Continuous in mmol/mol |  |
| HDL cholesterol | 30760 | 0, 1† | HDL cholesterol | Continuous in mmol/L |  |
| Triglyceride | 30870 | 0, 1† | Triglycerides | Continuous in mmol/L |  |
| Glucose | 30740 | 0, 1† | Glucose | Continuous in mmol/L |  |
| *We coded responses “Do not know” and “Prefer not to answer” to non-informative (i.e., missing);  †Instance 0 = baseline assessment (3/2006-10/2010), Instance 1 = first follow-up assessment (8/2012-6/2013). Priority was given to Instance 1 data when available given closer temporal proximity before accelerometry  Abbreviation: LPA: light physical activity; WW: weekend warrior; WC: Waist circumference; BMI: Body Mass Index; CVD: cardiovascular disease; MI: myocardial infarction; SBP: systolic blood pressure; DBP: diastolic blood pressure; BP: blood pressure; HbA1c: Glycated haemoglobin; HDL: high density lipoprotein. | | | | | |

Table 2: Covariates ascertainment and descriptions for NHANES.

| **Variables** | **File name in NHANES** | **Variable Name in NHANES** | **Description** | **Coding** | **Notes*** | **Timing of covariate measurements** |
| --- | --- | --- | --- | --- | --- | --- |
| Age | Demographic Variables & Sample Weights | RIDAGEEX | Exam Age in Months - Recode | Continuous age  in years | Age = RIDAGEEX/12 | 2003-2004;  2005-2006 |
| Sex | Demographic Variables & Sample Weights | RIAGENDR | Gender | 1) Male  2) Female |  | 2003-2004;  2005-2006 |
| Waist circumference | Body Measures | BMXWAIST | Waist Circumference (cm) | Continuous in cm |  | 2003-2004;  2005-2006 |
| Smoking | Smoking - Cigarette/Tobacco Use - Adult | SMQ020 SMQ040 | Smoking status | 1) Never  2) Previous  3) Current | We consider anyone who responds “No” to the question of whether they have ever smoked 100 cigarettes in their life (SMQ020) to be “Never” smokers. Former smokers are those individuals who respond “Yes” to having ever smoked 100 cigarettes in their life, but currently smoke “Not at all” (SMQ040). Current smokers are those individuals who both respond “Yes” to having ever smoked 100 cigarettes in their life and currently smoke either “Every day”, or “Some days” (SMQ040). | 2003-2004;  2005-2006 |
| Antihypertension medication | Prescription Medications | RXDDRGID | Generic drug code | 1) No  2) Yes | Any individual using “ANTIHYPERTENSIVE” medications is coded as “Yes”. | 2003-2004;  2005-2006 |
| Blood pressure class | Medical Conditions | BPQ020 | Ever told you had high blood pressure | 1) elevated  2) hypertension | SBP = (BPXSY1+BPXSY2+BPXSY3+BPXSY4)/4; DBP = (BPXDI1+BPXDI2+BPXDI3+BPXDI4)/4; Participants with SBP between 120 and 139 mmHg or DBP between 70 and 89 mmHg who were not using antihypertensive medication were classified as elevated; others were hypertension. | 2003-2004;  2005-2006 |
|  | Blood Pressure | BPXSY1  BPXSY2  BPXSY3  BPXSY4  BPXDI1  BPXDI2  BPXDI3  BPXDI4 | Systolic: Blood pressure (1-4 readings) mm Hg; Diastolic: Blood pressure (1-4 readings) mm Hg |  |  | 2003-2004;  2005-2006 |
| Cancer | Medical Conditions | MCQ220 | Ever told you had cancer or malignancy | 1) No  2) Yes | Any individual using “ANTINEOPLASTIC” medications or self-reporting a cancer diagnosis is coded as “Yes”. | 2003-2004;  2005-2006 |
|  | Prescription Medications | RXDDRGID | Generic drug code |  |  | 2003-2004;  2005-2006 |
| Diabetes | Medical Conditions | DIQ010 | Doctor told you have diabetes | 1) No  2) Yes | Any individual using “ANTIDIABETIC” medications or self-reporting a diabetes diagnosis is coded as “Yes”. | 2003-2004;  2005-2006 |
|  | Prescription Medications | RXDDRGID | Generic drug code |  |  | 2003-2004;  2005-2006 |
| MI | Medical Conditions | MCQ160E | Ever told you had heart attack | 1) No  2) Yes |  | 2003-2004;  2005-2006 |
| stroke | Medical Conditions | MCQ160F | Ever told you had a stroke | 1) No  2) Yes |  | 2003-2004;  2005-2006 |
| Glucose | Plasma Fasting Glucose, Serum C-peptide & Insulin | LBXGLU | Glucose, plasma (mg/dL) | Continuous in mmol/L | Glucose = LBXGLU/18 | 2003-2004;  2005-2006 |
| HbA1c | Glycohemoglobin | LBXGH | Glycohemoglobin (%) | Continuous in mmol/mol | HbA1c=10.93 *Glycohemoglobin- 23.50 | 2003-2004;  2005-2006 |
| Sedentary time | Physical Activity Monitor | PAXSTAT  PAXCAL  PAXDAY  PAXHOUR  PAXINTEN |  | Continuous in hours/week | See details in “Exposure ascertainment” of the method section. | 2003-2004;  2005-2006 |
| *We coded responses “Refused” and “Don't know” to non-informative (i.e., missing). | | | | | | |
