# Supplementary material for: Physical Activity Recommendations Tailored by a Predictive Model for Adults With High Blood Pressure: Observational Study
Source: J Med Internet Res. 2026 Jan 9;28:e78492. doi: 10.2196/78492 (PMC12788716; doi:10.2196/78492)
Supplement: Multimedia Appendix 3 [file jmir-v28-e78492-s003.docx]

**Multimedia Appendix 4.** The reliability measurements of covariates

| **Variable** | **Type** | **Number** | **Proportion (%)** | **Consistency Metric** | **Metric Value** | **Metric 95%CI** |
| --- | --- | --- | --- | --- | --- | --- |
| BMI (Kg/m^2^) | continuous | 7,253 | 10.12 | ICC | 0.927 | [0.923, 0.930] |
| Waist circumference (cm) | continuous | 7,257 | 10.13 | ICC | 0.857 | [0.834, 0.876] |
| SBP (mmHg) | continuous | 7,263 | 10.14 | ICC | 0.631 | [0.613, 0.649] |
| DBP (mmHg) | continuous | 7,263 | 10.14 | ICC | 0.608 | [0.589, 0.625] |
| HbA1c (mmol/mol) | continuous | 4,608 | 6.43 | ICC | 0.738 | [0.713, 0.761] |
| HDL cholesterol (mmol/L) | continuous | 4,956 | 6.92 | ICC | 0.842 | [0.797, 0.874] |
| Triglyceride (mmol/L) | continuous | 6,048 | 8.44 | ICC | 0.615 | [0.599, 0.630] |
| Glucose (mmol/L) | continuous | 4,949 | 6.91 | ICC | 0.393 | [0.369, 0.416] |
| Education | categorical | 7,092 | 9.90 | Cohen’s Kappa | 0.829 | / |
| Smoking | categorical | 7,255 | 10.13 | Cohen’s Kappa | 0.881 | / |
| Alcohol | categorical | 7,266 | 10.14 | Cohen’s Kappa | 0.703 | / |
| Added salt | categorical | 7,268 | 10.15 | Cohen’s Kappa | 0.518 | / |
| Antihypertension medication | categorical | 7,108 | 9.92 | Cohen’s Kappa | 0.777 | / |
| Family CVD | categorical | 7,230 | 10.09 | Cohen’s Kappa | 0.672 | / |

Note: The intraclass correlation coefficient (ICC) was calculated based on a two-way mixed-effects model with an absolute agreement definition for single measurements. Cohen’s kappa was computed using an unweighted approach. Abbreviation: WC: Waist circumference; BMI: Body Mass Index; CVD: cardiovascular disease; MI: myocardial infarction; SBP: systolic blood pressure; DBP: diastolic blood pressure; BP: blood pressure; HbA1c: Glycated haemoglobin; HDL: high density lipoprotein.
