# Supplementary figures and images for: Physical Activity Recommendations Tailored by a Predictive Model for Adults With High Blood Pressure: Observational Study
Source: J Med Internet Res. 2026 Jan 9;28:e78492. doi: 10.2196/78492 (PMC12788716; doi:10.2196/78492)

**Multimedia Appendix 1.** Study flow for NHANES


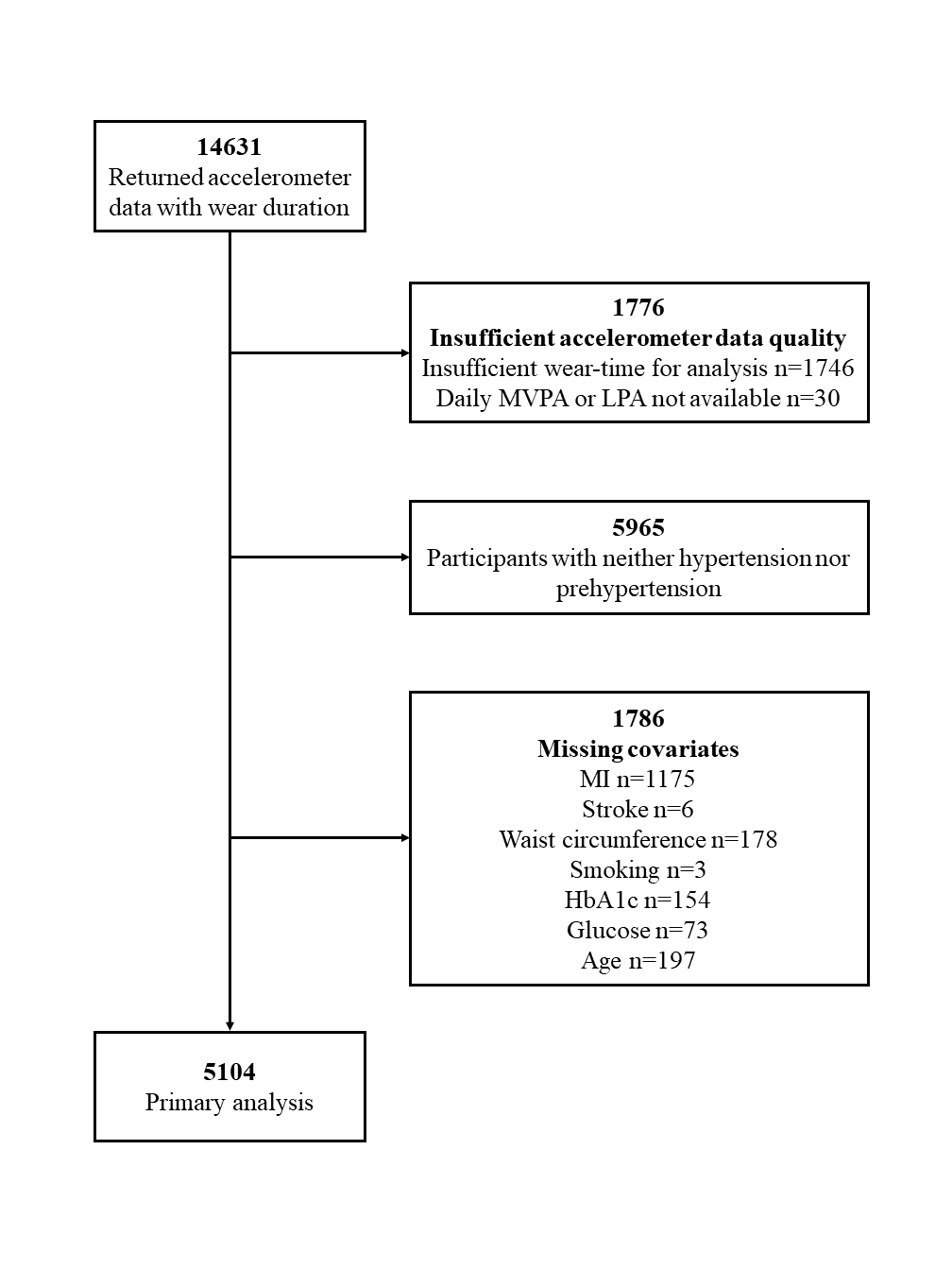

Supplement: Multimedia Appendix 4 [file jmir-v28-e78492-s004.docx]
