# Supplementary material for: Physical Activity Recommendations Tailored by a Predictive Model for Adults With High Blood Pressure: Observational Study
Source: J Med Internet Res. 2026 Jan 9;28:e78492. doi: 10.2196/78492 (PMC12788716; doi:10.2196/78492)

**Multimedia Appendix 5.** Time to censoring and time to death for the UKB and NHANES sets

A) time to censoring and time to death for UK Biobank training sets: median time to death was 6.1 years; median time to censoring was 9.5 years;

B) time to censoring and time to death for NHANES validation sets: median time to death was 8.7 years; median time to censoring was 14.8 years.


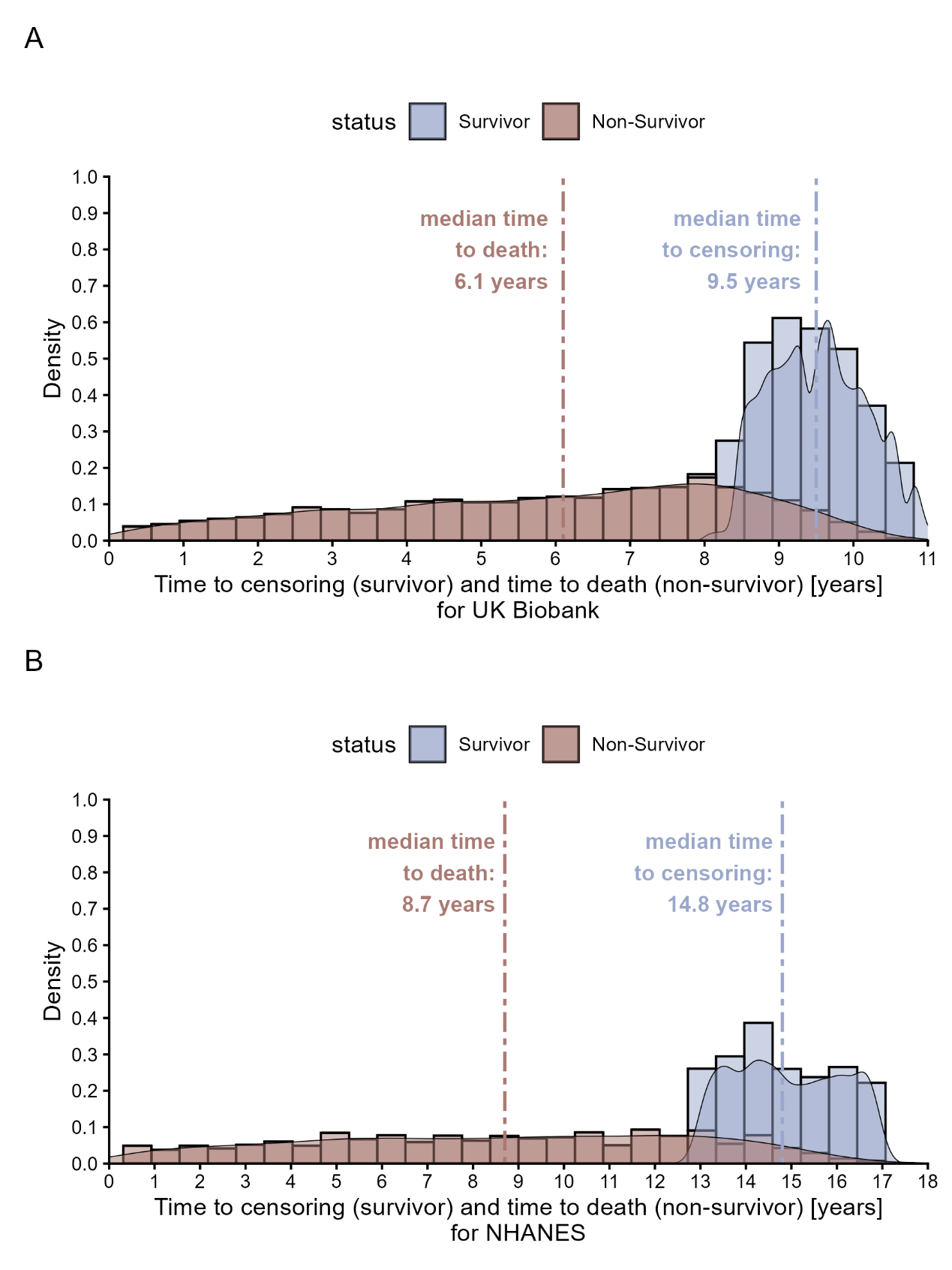

Supplement: Multimedia Appendix 5 [file jmir-v28-e78492-s005.docx]
