# Supplementary material for: Physical Activity Recommendations Tailored by a Predictive Model for Adults With High Blood Pressure: Observational Study
Source: J Med Internet Res. 2026 Jan 9;28:e78492. doi: 10.2196/78492 (PMC12788716; doi:10.2196/78492)
Supplement: Multimedia Appendix 6 [file jmir-v28-e78492-s006.docx]

**Multimedia Appendix 6.** Baseline characteristics of participants

Table 1: Baseline characteristics of participants for the UKB cohort.

| Variables | Levels | Baseline PA (N=12577) | Active LPA (N=12782) | Active regular (N=15141) | Active WW (N=31137) | *p* |
| --- | --- | --- | --- | --- | --- | --- |
| Sedentary time | Median (IQR) | 72.5 (61.4 to 81.1) | 61.2 (53.5 to 68.1) | 61.7 (53.0 to 70.1) | 62.6 (53.9 to 71.0) | <.001 |
| Sleep time | Median (IQR) | 60.1 (53.0 to 67.4) | 58.8 (53.9 to 63.7) | 58.7 (53.9 to 63.6) | 59.0 (53.9 to 64.3) | <.001 |
| Age (year) | Median (IQR) | 65.0 (57.6 to 69.8) | 64.9 (57.9 to 69.4) | 62.4 (55.4 to 67.8) | 63.7 (56.8 to 68.5) | <.001 |
| Sex | Male | 5693 (45.3%) | 3202 (25.1%) | 7821 (51.7%) | 16155 (51.9%) | <.001 |
|  | Female | 6884 (54.7%) | 9580 (74.9%) | 7320 (48.3%) | 14982 (48.1%) |  |
| Education | School leaver | 3422 (27.2%) | 3958 (31%) | 2963 (19.6%) | 6351 (20.4%) | <.001 |
|  | Further education | 4538 (36.1%) | 4659 (36.4%) | 4707 (31.1%) | 10297 (33.1%) |  |
|  | Higher education | 4617 (36.7%) | 4165 (32.6%) | 7471 (49.3%) | 14489 (46.5%) |  |
| Ethnicity | White | 12209 (97.1%) | 12338 (96.5%) | 14713 (97.2%) | 30406 (97.7%) | <.001 |
|  | Nonwhite | 368 (2.9%) | 444 (3.5%) | 428 (2.8%) | 731 (2.3%) |  |
| WC (cm) | Median (IQR) | 94.0 (84.0 to 103.0) | 87.2 (79.0 to 97.0) | 87.0 (78.0 to 96.0) | 88.0 (79.0 to 97.0) | <.001 |
| BMI (Kg/m^2^) | Median (IQR) | 28.1 (25.3 to 31.8) | 26.7 (24.1 to 30.0) | 25.5 (23.2 to 28.1) | 25.9 (23.6 to 28.5) | <.001 |
| Smoking | Never | 6535 (52%) | 7269 (56.9%) | 8730 (57.7%) | 18299 (58.8%) | <.001 |
|  | Previous | 4830 (38.4%) | 4598 (36%) | 5532 (36.5%) | 11164 (35.9%) |  |
|  | Current | 1212 (9.6%) | 915 (7.2%) | 879 (5.8%) | 1674 (5.4%) |  |
| Alcohol | <3 times/week | 7143 (56.8%) | 7358 (57.6%) | 7144 (47.2%) | 14656 (47.1%) | <.001 |
|  | 3+ times/week | 5434 (43.2%) | 5424 (42.4%) | 7997 (52.8%) | 16481 (52.9%) |  |
| Added salt | Never/rarely | 7190 (57.2%) | 7366 (57.6%) | 9403 (62.1%) | 18922 (60.8%) | <.001 |
|  | Sometimes | 3396 (27%) | 3556 (27.8%) | 3833 (25.3%) | 8191 (26.3%) |  |
|  | Usually | 1499 (11.9%) | 1420 (11.1%) | 1480 (9.8%) | 3141 (10.1%) |  |
|  | Always | 492 (3.9%) | 440 (3.4%) | 425 (2.8%) | 883 (2.8%) |  |
| Family CVD | No | 2833 (22.5%) | 2735 (21.4%) | 3690 (24.4%) | 7565 (24.3%) | <.001 |
|  | Yes | 9744 (77.5%) | 10047 (78.6%) | 11451 (75.6%) | 23572 (75.7%) |  |
| Cancer | No | 11281 (89.7%) | 11614 (90.9%) | 13975 (92.3%) | 28632 (92%) | <.001 |
|  | Yes | 1296 (10.3%) | 1168 (9.1%) | 1166 (7.7%) | 2505 (8%) |  |
| Diabetes | No | 11329 (90.1%) | 12111 (94.8%) | 14611 (96.5%) | 29991 (96.3%) | <.001 |
|  | Yes | 1248 (9.9%) | 671 (5.2%) | 530 (3.5%) | 1146 (3.7%) |  |
| MI | No | 12063 (95.9%) | 12515 (97.9%) | 14850 (98.1%) | 30525 (98%) | <.001 |
|  | Yes | 514 (4.1%) | 267 (2.1%) | 291 (1.9%) | 612 (2%) |  |
| Stroke | No | 12242 (97.3%) | 12583 (98.4%) | 14965 (98.8%) | 30731 (98.7%) | <.001 |
|  | Yes | 335 (2.7%) | 199 (1.6%) | 176 (1.2%) | 406 (1.3%) |  |
| Antihypertension medication | No | 9132 (72.6%) | 10113 (79.1%) | 12831 (84.7%) | 26019 (83.6%) | <.001 |
|  | Yes | 3445 (27.4%) | 2669 (20.9%) | 2310 (15.3%) | 5118 (16.4%) |  |
| SBP (mmHg) | Median (IQR) | 138.0 (127.0 to 150.5) | 137.0 (126.5 to 150.0) | 136.0 (125.5 to 148.0) | 136.5 (126.0 to 149.0) | <.001 |
| DBP (mmHg) | Median (IQR) | 83.0 (77.0 to 89.5) | 81.5 (76.0 to 88.5) | 82.0 (76.0 to 88.5) | 82.0 (76.0 to 88.5) | <.001 |
| BP class | Elevated | 4738 (37.7%) | 5684 (44.5%) | 7530 (49.7%) | 14927 (47.9%) | <.001 |
|  | Hypertension | 7839 (62.3%) | 7098 (55.5%) | 7611 (50.3%) | 16210 (52.1%) |  |
| HbA1c (mmol/mol) | Median (IQR) | 35.3 (32.9 to 38.3) | 35.4 (33.0 to 37.9) | 34.7 (32.3 to 37.1) | 34.8 (32.5 to 37.1) | <.001 |
| HDL cholesterol (mmol/L) | Median (IQR) | 1.3 (1.1 to 1.6) | 1.5 (1.2 to 1.8) | 1.5 (1.2 to 1.8) | 1.4 (1.2 to 1.7) | <.001 |
| Triglyceride (mmol/L) | Median (IQR) | 1.6 (1.2 to 2.3) | 1.5 (1.1 to 2.1) | 1.3 (1.0 to 1.9) | 1.4 (1.0 to 2.0) | <.001 |
| Glucose (mmol/L) | Median (IQR) | 5.0 (4.6 to 5.4) | 4.9 (4.6 to 5.3) | 4.9 (4.6 to 5.3) | 4.9 (4.6 to 5.3) | <.001 |
| Best pattern predicted | Inactive | 190 (1.5%) | 115 (0.9%) | 108 (0.7%) | 216 (0.7%) | <.001 |
|  | Active LPA | 3146 (25%) | 4345 (34%) | 5238 (34.6%) | 9995 (32.1%) |  |
|  | Active regular | 5367 (42.7%) | 5032 (39.4%) | 5500 (36.3%) | 11390 (36.6%) |  |
|  | Active WW | 3874 (30.8%) | 3290 (25.7%) | 4295 (28.4%) | 9536 (30.6%) |  |

Abbreviation: LPA: light physical activity; WW: weekend warrior; WC: Waist circumference; BMI: Body Mass Index; CVD: cardiovascular disease; MI: myocardial infarction; SBP: systolic blood pressure; DBP: diastolic blood pressure; BP: blood pressure; HbA1c: Glycated haemoglobin; HDL: high density lipoprotein.

Table 2: Baseline characteristics of participants for the NHANES cohort.

| Variables | Levels | Baseline PA (N=1825) | Active LPA (N=1753) | Active regular (N=810) | Active WW (N=716) | p |
| --- | --- | --- | --- | --- | --- | --- |
| Age | Median (IQR) | 61.3 (44.0 to 73.2) | 58.5 (45.7 to 68.6) | 45.3 (34.8 to 55.1) | 43.7 (33.8 to 55.4) | <.001 |
| Sex | Male | 881 (48.3%) | 733 (41.8%) | 582 (71.9%) | 506 (70.7%) | <.001 |
|  | Female | 944 (51.7%) | 1020 (58.2%) | 228 (28.1%) | 210 (29.3%) |  |
| WC (cm) | Median (IQR) | 102.5 (92.7 to 113.5) | 100.0 (90.5 to 109.6) | 95.5 (87.1 to 104.2) | 97.0 (87.8 to 105.1) | <.001 |
| Smoking | Never | 836 (45.8%) | 897 (51.2%) | 426 (52.6%) | 382 (53.4%) | <.001 |
|  | Previous | 581 (31.8%) | 518 (29.5%) | 236 (29.1%) | 175 (24.4%) |  |
|  | Current | 408 (22.4%) | 338 (19.3%) | 148 (18.3%) | 159 (22.2%) |  |
| Sedentary time (hour/week) | Median (IQR) | 55.3 (40.8 to 70.1) | 54.7 (44.5 to 64.7) | 49.5 (40.0 to 61.4) | 46.3 (35.9 to 58.3) | <.001 |
| Antihypertension medication | No | 1652 (90.5%) | 1574 (89.8%) | 776 (95.8%) | 681 (95.1%) | <.001 |
|  | Yes | 173 (9.5%) | 179 (10.2%) | 34 (4.2%) | 35 (4.9%) |  |
| BP class | Elevated blood pressure | 626 (34.3%) | 690 (39.4%) | 489 (60.4%) | 433 (60.5%) | <.001 |
|  | Hypertension | 1199 (65.7%) | 1063 (60.6%) | 321 (39.6%) | 283 (39.5%) |  |
| MI | No | 1675 (91.8%) | 1676 (95.6%) | 792 (97.8%) | 698 (97.5%) | <.001 |
|  | Yes | 150 (8.2%) | 77 (4.4%) | 18 (2.2%) | 18 (2.5%) |  |
| Stroke | No | 1694 (92.8%) | 1697 (96.8%) | 800 (98.8%) | 707 (98.7%) | <.001 |
|  | Yes | 131 (7.2%) | 56 (3.2%) | 10 (1.2%) | 9 (1.3%) |  |
| Diabetes | No | 938 (51.4%) | 955 (54.5%) | 521 (64.3%) | 462 (64.5%) | <.001 |
|  | Yes | 887 (48.6%) | 798 (45.5%) | 289 (35.7%) | 254 (35.5%) |  |
| Cancer | No | 1559 (85.4%) | 1561 (89%) | 761 (94%) | 678 (94.7%) | <.001 |
|  | Yes | 266 (14.6%) | 192 (11%) | 49 (6%) | 38 (5.3%) |  |
| Glucose (mmol/L) | Median (IQR) | 5.3 (4.8 to 6.1) | 5.2 (4.8 to 5.8) | 5.1 (4.7 to 5.4) | 5.1 (4.7 to 5.5) | <.001 |
| HbA1c (mmol/mol) | Median (IQR) | 36.6 (33.3 to 42.1) | 36.6 (33.3 to 41.0) | 34.4 (32.2 to 37.7) | 35.5 (32.2 to 37.7) | <.001 |
| Best pattern predicted | inactive | 54 (3%) | 23 (1.3%) | 4 (0.5%) | 3 (0.4%) | <.001 |
|  | active LPA | 659 (36.1%) | 742 (42.3%) | 504 (62.2%) | 469 (65.5%) |  |
|  | active regular | 429 (23.5%) | 498 (28.4%) | 196 (24.2%) | 152 (21.2%) |  |
|  | active WW | 683 (37.4%) | 490 (28%) | 106 (13.1%) | 92 (12.8%) |  |

Abbreviation: LPA: light physical activity; WW: weekend warrior; WC: Waist circumference; MI: myocardial infarction; BP: blood pressure; HbA1c: Glycated haemoglobin.
