# Supplementary material for: Physical Activity Recommendations Tailored by a Predictive Model for Adults With High Blood Pressure: Observational Study
Source: J Med Internet Res. 2026 Jan 9;28:e78492. doi: 10.2196/78492 (PMC12788716; doi:10.2196/78492)

**Multimedia Appendix 7.** The detailed results obtained using the LASSO penalized Cox model for covariate selection

Variables initially included in the model are: PA patterns, age, sex, ethnicity, body mass index (BMI), waist circumference, education, smoking status, alcohol, added salt, sedentary time, sleep time, SBP, DBP, antihypertension medication, cancer, diabetes, myocardial infarction (MI), stroke, family cardiovascular disease (CVD), blood pressure class, Glycated hemoglobin (HbA1c), high density lipoprotein cholesterol, triglyceride, and glucose. Variables selected are: PA patterns, age, sex, sedentary time, smoking status, antihypertension medication, cancer, diabetes, MI, stroke, blood pressure class, waist circumference, glucose, and HbA1c. While *Glucose* was initially selected by LASSO, it was subsequently removed during the backward elimination process due to concerns regarding its reliability and limited contribution to the predictive model.

**Table 1.** Variables considered for the selection based on the Cox model with LASSO penalization

| **Covariates** | **Levels** | **Coefficient** |
| --- | --- | --- |
| Physical activity patterns | Active LPA vs baseline PA | 0 |
|  | Active regular vs baseline PA | 0 |
|  | Active WW vs baseline PA | -0.084 |
| Sex | Female vs male | -0.180 |
| Age (year) | / | 0.084 |
| Sedentary time (hours/week) | / | 0.006 |
| SBP (mmHg) | / | 0 |
| DBP (mmHg) | / | 0 |
| Smoking status | Previous vs never | 0.107 |
|  | Current vs never | 0.531 |
| Education | Further education vs school leaver | 0 |
|  | Higher education vs school leaver | 0 |
| BP class | Hypertension vs elevated | 0.076 |
| Sleep time (hour/week) | / | 0 |
| Antihypertension medication | Yes vs no | 0.143 |
| Diabetes | Yes vs no | 0.141 |
| Cancer | Yes vs no | 0.538 |
| MI | Yes vs no | 0.336 |
| Stroke | Yes vs no | 0.306 |
| Ethnicity | Nonwhite vs white | 0 |
| BMI (Kg/m2) | / | 0 |
| Alcohol | 3+ times/week vs <3 times/week | 0 |
| Family CVD | Yes vs no | 0 |
| Waist circumference (cm) | / | 0.010 |
| HbA1c (mmol/mol) | / | 0.010 |
| HDL cholesterol (mmol/L) | / | 0 |
| Glucose (mmol/L) | / | 0.007 |
| Triglyceride (mmol/L) | / | 0 |
| Added salt | Sometimes vs never | 0 |
|  | Usually vs never | 0 |
|  | Always vs never | 0 |
| Abbreviation: LPA: light physical activity; WW: weekend warrior; BMI: Body Mass Index; CVD: cardiovascular disease; MI: myocardial infarction; SBP: systolic blood pressure; DBP: diastolic blood pressure; BP: blood pressure; HbA1c: Glycated haemoglobin; HDL: high density lipoprotein. | | |

**Figure 1.** Lasso model for variable selection

A) paths of the coefficients as a function of the log lambda

B) cross-validated deviance curve as a function of the shrinkage parameter lambda

A.


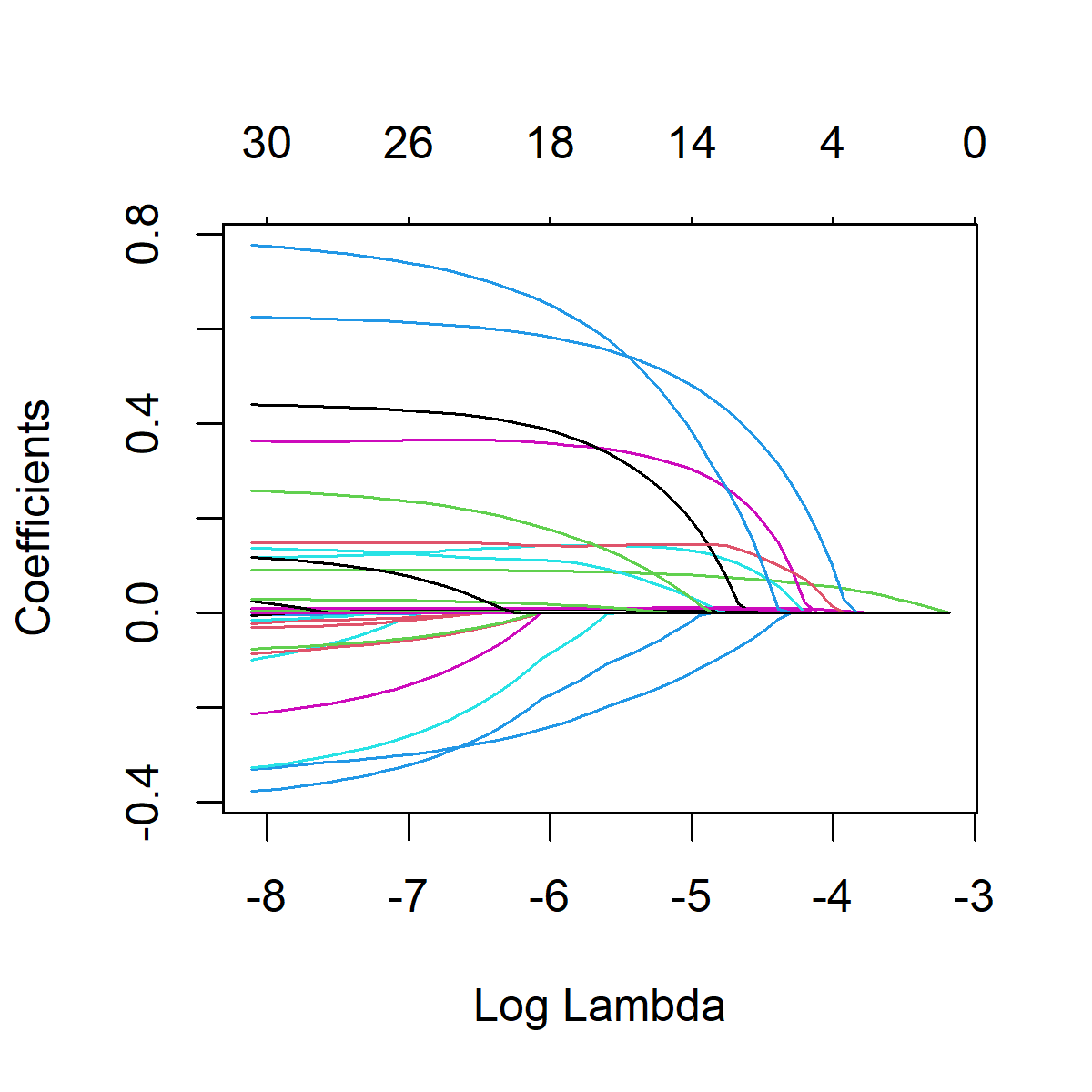


B.


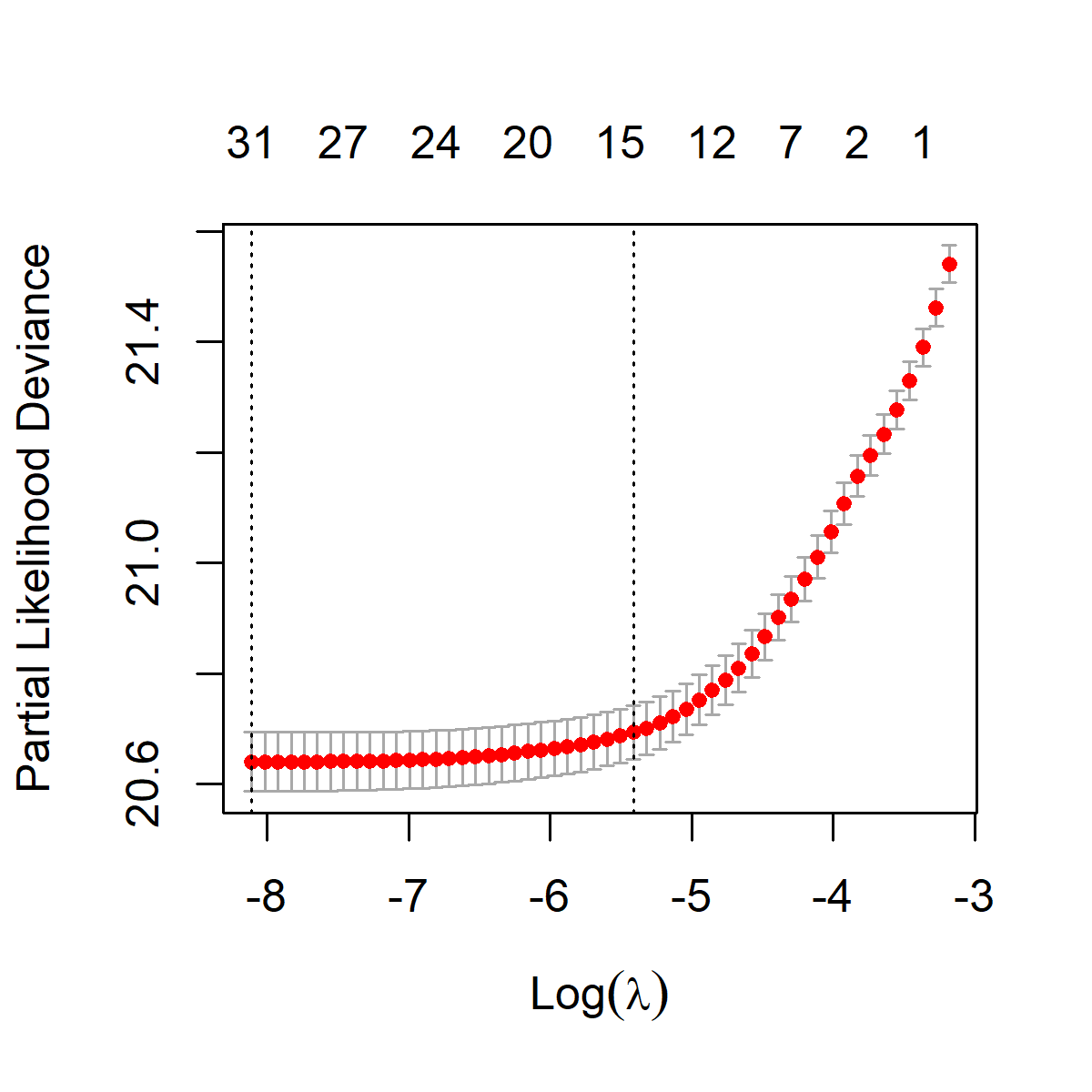

Supplement: Multimedia Appendix 7 [file jmir-v28-e78492-s007.docx]
