# Supplementary material for: Physical Activity Recommendations Tailored by a Predictive Model for Adults With High Blood Pressure: Observational Study
Source: J Med Internet Res. 2026 Jan 9;28:e78492. doi: 10.2196/78492 (PMC12788716; doi:10.2196/78492)
Supplement: Multimedia Appendix 8 [file jmir-v28-e78492-s008.docx]

**Multimedia Appendix 8.** The associations between covariates selected and all-cause mortality by univariate Cox models in the UKB cohort

| **Covariates** | **Levels** | **HR**  **(95%CI)** | ***p*-value** |
| --- | --- | --- | --- |
| Physical activity patterns | Active LPA vs Baseline PA | 0.56 (0.51, 0.62) | <.001 |
|  | Active regular vs Baseline PA | 0.45 (0.41, 0.50) | <.001 |
|  | Active WW vs Baseline PA | 0.47 (0.43, 0.51) | <.001 |
| Sex | Female vs Male | 0.53 (0.50, 0.57) | <.001 |
| Age (year) | / | 1.12 (1.11, 1.13) | <.001 |
| Sedentary time (hours/week) | / | 1.02 (1.02, 1.02) | <.001 |
| Smoking status | Previous vs Never | 1.76 (1.64, 1.89) | <.001 |
|  | Current vs Never | 2.32 (2.07, 2.60) | <.001 |
| BP class | Hypertension vs Elevated | 2.30 (2.13, 2.48) | <.001 |
| Antihypertension medication | Yes vs No | 2.38 (2.22, 2.56) | <.001 |
| Diabetes | Yes vs No | 2.70 (2.44, 3.00) | <.001 |
| Cancer | Yes vs No | 2.66 (2.45, 2.90) | <.001 |
| MI | Yes vs No | 3.33 (2.92, 3.80) | <.001 |
| stroke | Yes vs No | 2.91 (2.45, 3.44) | <.001 |
| Waist circumference (cm) | / | 1.03 (1.03, 1.03) | <.001 |
| HbA1c (mmol/mol) | / | 1.04 (1.04, 1.04) | <.001 |
| Glucose (mmol/L) | / | 1.15 (1.13, 1.17) | <.001 |
| Abbreviation: LPA: light physical activity; WW: weekend warrior; BP: blood pressure; MI: myocardial infarction; HbA1c: Glycated haemoglobin; HR: hazard ratio; CI: confidence interval. | | | |
