# Supplementary material for: Physical Activity Recommendations Tailored by a Predictive Model for Adults With High Blood Pressure: Observational Study
Source: J Med Internet Res. 2026 Jan 9;28:e78492. doi: 10.2196/78492 (PMC12788716; doi:10.2196/78492)
Supplement: Multimedia Appendix 9 [file jmir-v28-e78492-s009.docx]

**Multimedia Appendix 9**. Coefficients and standard errors of the Cox prediction model

Table 1. Coefficients and standard errors of the Cox prediction model.

| Features | Estimated coefficient (SE) | Features | Estimated coefficient (SE) |
| --- | --- | --- | --- |
| Active LPA vs baseline PA | -1.655 (0.741) | Active LPA * Diabetes | -0.214 (0.206) |
| Active regular vs baseline PA | -0.830 (0.706) | Active regular * Diabetes | 0.236 (0.208) |
| Active WW vs baseline PA | -0.231 (0.584) | Active WW * Diabetes | -0.170 (0.177) |
| Female | -0.258 (0.068) | Active LPA * MI | 0.314 (0.199) |
| Age | 0.091 (0.006) | Active regular * MI | 0.119 (0.215) |
| Sedentary time | 0.008 (0.002) | Active WW * MI | 0.125 (0.170) |
| Cancer: yes vs no | 0.564 (0.078) | Active LPA * Stroke | 0.590 (0.253) |
| Diabetes: yes vs no | 0.198 (0.111) | Active regular * Stroke | 0.625 (0.260) |
| MI: yes vs no | 0.262 (0.111) | Active WW * Stroke | 0.476 (0.217) |
| Stroke: yes vs no | 0.124 (0.152) | Active LPA * Antihypertension medication | -0.091 (0.124) |
| Antihypertension medication | 0.105 (0.076) | Active regular * Antihypertension medication | 0.165 (0.131) |
| BP class | 0.123 (0.087) | Active WW * Antihypertension medication | 0.048 (0.104) |
| Waist circumference | 0.008 (0.002) | Active LPA * BP class | 0.199 (0.137) |
| HbA1c | 0.009 (0.005) | Active regular * BP class | -0.111 (0.134) |
| Smoking: previous vs never | 0.263 (0.037) | Active WW * BP class | 0.081 (0.111) |
| Smoking: current vs never | 0.776 (0.059) | Active LPA * Cancer | 0.141 (0.129) |
| Active LPA * Female | -0.215 (0.109) | Active regular * Cancer | -0.027 (0.139) |
| Active regular * Female | -0.126 (0.115) | Active WW * Cancer | 0.123 (0.108) |
| Active WW * Female | -0.015 (0.091) | Active LPA * HbA1c | 0.011 (0.008) |
| Active LPA * Age | 0.014 (0.010) | Active regular * HbA1c | -0.001 (0.009) |
| Active regular * Age | 0.011 (0.009) | Active WW * HbA1c | 0.008 (0.007) |
| Active WW * Age | -0.007 (0.007) | Active regular * Sedentary time | -0.004 (0.004) |
| Active LPA * Sedentary time | -0.001 (0.004) | Active WW * Sedentary time | -0.002 (0.003) |
| Note: Variables selected by Lasso are: PA patterns, age, sex, sedentary time, smoking status, antihypertension medication, cancer, diabetes, MI, stroke, blood pressure class, waist circumference, glucose, and HbA1c. The original predictive model incorporated all second-order interactions of PA patterns with each selected covariate. Subsequently, following stepwise backward elimination, the main effect of glucose and interactions of PA patterns with waist circumference, smoking status, and glucose were removed from the model. Abbreviation: LPA: light physical activity; WW: weekend warrior; BP: blood pressure; MI: myocardial infarction; HbA1c: Glycated haemoglobin. | | | |
