# Supplementary material for: Physical Activity Recommendations Tailored by a Predictive Model for Adults With High Blood Pressure: Observational Study
Source: J Med Internet Res. 2026 Jan 9;28:e78492. doi: 10.2196/78492 (PMC12788716; doi:10.2196/78492)
Supplement: Multimedia Appendix 10 [file jmir-v28-e78492-s010.docx]

**Multimedia Appendix 10.** Snapshots from the web app

Figure 1: The English version of the web app (https://yyhget.shinyapps.io/Rshiny_hyper_en/)

**
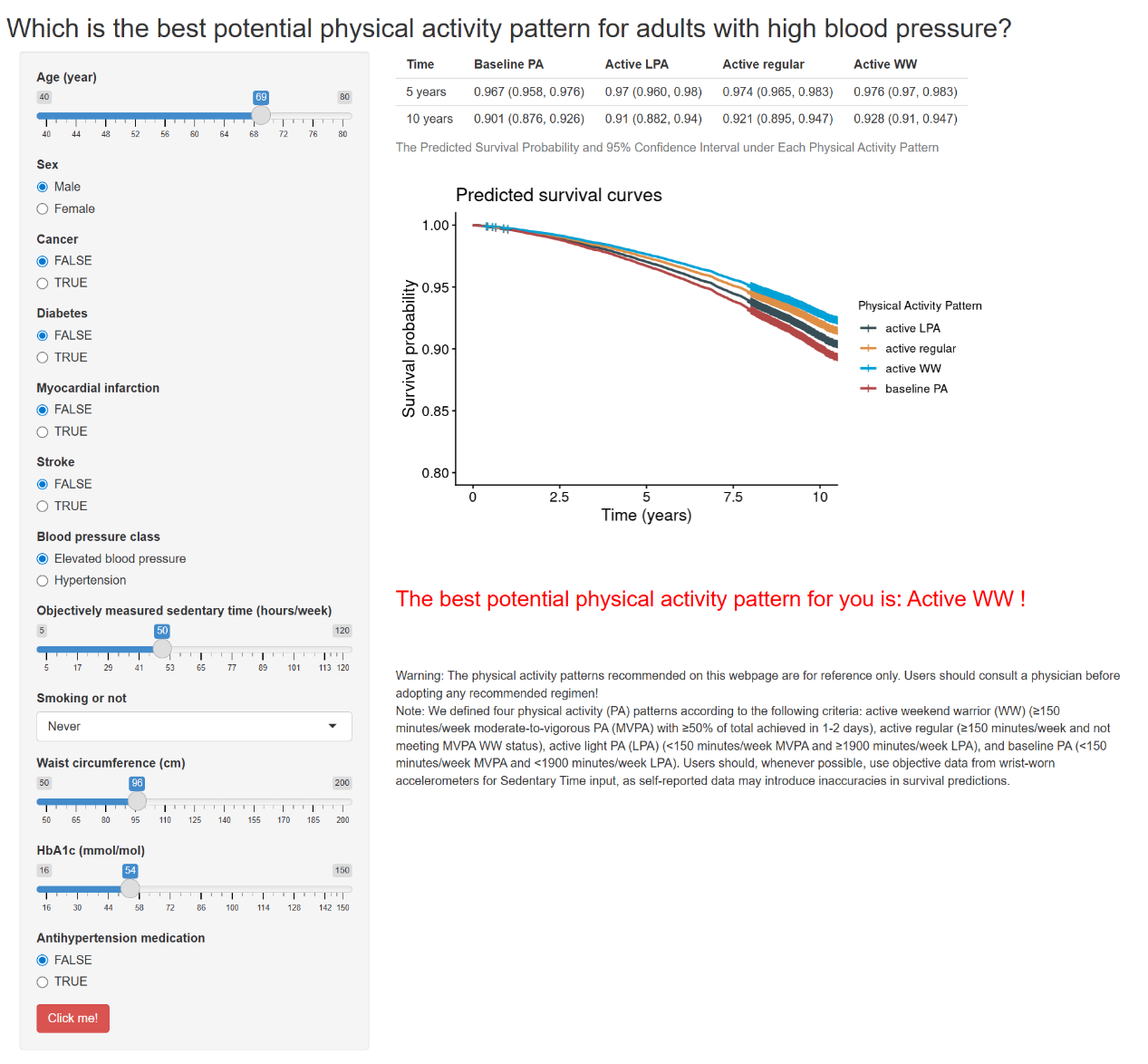
**

Note: Users need enter the required clinical characteristics (including age, sex, sedentary time, smoking status, antihypertension medication, cancer, diabetes, MI, stroke, blood pressure class, waist circumference, and HbA1c) into the website. The tool then calculates the predicted 5-year and 10-year survival probabilities predictions corresponding to four physical activity patterns. After these probabilities are generated, the application identifies the pattern with the highest predicted survival. This information can support clinicians and patients in selecting the most appropriate personalized physical activity pattern.

Figure 2: The Chinese version of the web app (https://yyhget.shinyapps.io/Rshiny_hyper_cn/)


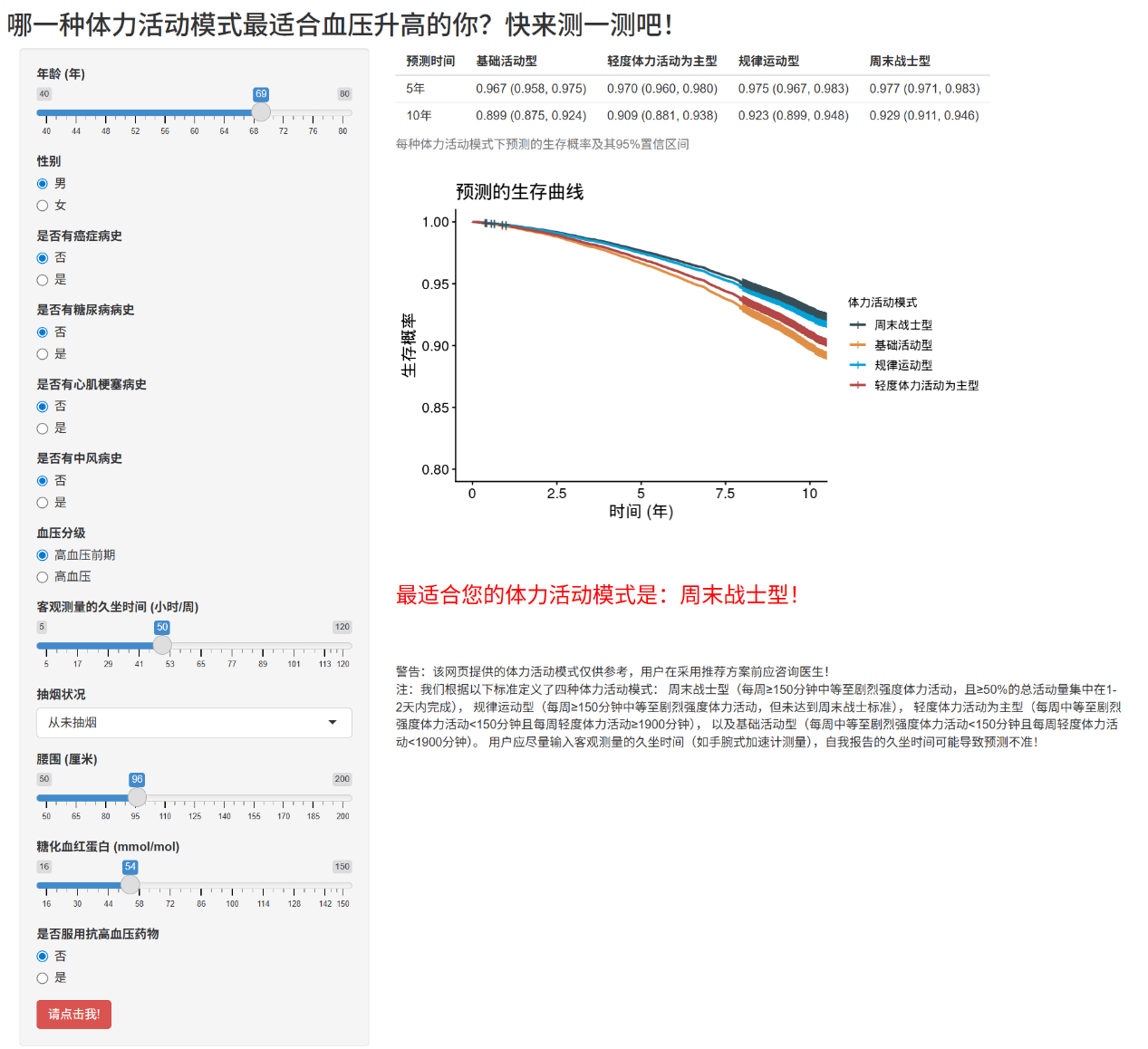


Note: Users need enter the required clinical characteristics (including age, sex, sedentary time, smoking status, antihypertension medication, cancer, diabetes, MI, stroke, blood pressure class, waist circumference, and HbA1c) into the website. The tool then calculates the predicted 5-year and 10-year survival probabilities predictions corresponding to four physical activity patterns. After these probabilities are generated, the application identifies the pattern with the highest predicted survival. This information can support clinicians and patients in selecting the most appropriate personalized physical activity pattern.
