# Supplementary material for: Physical Activity Recommendations Tailored by a Predictive Model for Adults With High Blood Pressure: Observational Study
Source: J Med Internet Res. 2026 Jan 9;28:e78492. doi: 10.2196/78492 (PMC12788716; doi:10.2196/78492)
Supplement: Multimedia Appendix 11 [file jmir-v28-e78492-s011.docx]

**Multimedia Appendix 11.** A baseline characteristic of participants stratified by the predicted optimal PA pattern in the NHANES cohort.

| Variables | Levels | Baseline PA (N=73) | Active LPA (N=2425) | Active regular (N=1219) | Active WW (N=1387) | *p* |
| --- | --- | --- | --- | --- | --- | --- |
| Pattern actually observed | Baseline PA | 45 (61.6%) | 662 (27.3%) | 428 (35.1%) | 690 (49.7%) | <.001 |
|  | Active LPA | 18 (24.7%) | 765 (31.5%) | 453 (37.2%) | 517 (37.3%) |  |
|  | Active regular | 6 (8.2%) | 521 (21.5%) | 184 (15.1%) | 99 (7.1%) |  |
|  | Active WW | 4 (5.5%) | 477 (19.7%) | 154 (12.6%) | 81 (5.8%) |  |
| Age | Median (IQR) | 66.5 (62.8 to 72.3) | 42.8 (32.0 to 51.5) | 54.2 (45.0 to 63.5) | 73.2 (67.8 to 78.6) | <.001 |
| Sex | Male | 45 (61.6%) | 1135 (46.8%) | 648 (53.2%) | 874 (63%) | <.001 |
|  | Female | 28 (38.4%) | 1290 (53.2%) | 571 (46.8%) | 513 (37%) |  |
| WC (cm) | Median (IQR) | 108.0 (97.0 to 117.6) | 96.6 (87.4 to 107.2) | 102.1 (93.1 to 112.7) | 101.4 (93.0 to 110.0) | <.001 |
| Smoking | Never | 25 (34.2%) | 1348 (55.6%) | 581 (47.7%) | 587 (42.3%) | <.001 |
|  | Previous | 31 (42.5%) | 523 (21.6%) | 326 (26.7%) | 630 (45.4%) |  |
|  | Current | 17 (23.3%) | 554 (22.8%) | 312 (25.6%) | 170 (12.3%) |  |
| Sedentary time (hours/week) | Median (IQR) | 58.4 (47.3 to 67.0) | 47.0 (36.5 to 57.4) | 56.0 (43.2 to 68.8) | 61.2 (50.8 to 71.9) | <.001 |
| Antihypertension medication | No | 63 (86.3%) | 2236 (92.2%) | 1162 (95.3%) | 1222 (88.1%) | <.001 |
|  | Yes | 10 (13.7%) | 189 (7.8%) | 57 (4.7%) | 165 (11.9%) |  |
| Blood pressure class | Elevated | 4 (5.5%) | 1788 (73.7%) | 138 (11.3%) | 308 (22.2%) | <.001 |
|  | Hypertension | 69 (94.5%) | 637 (26.3%) | 1081 (88.7%) | 1079 (77.8%) |  |
| MI | No | 49 (67.1%) | 2410 (99.4%) | 1160 (95.2%) | 1222 (88.1%) | <.001 |
|  | Yes | 24 (32.9%) | 15 (0.6%) | 59 (4.8%) | 165 (11.9%) |  |
| Stroke | No | 0 (0%) | 2403 (99.1%) | 1203 (98.7%) | 1292 (93.2%) | <.001 |
|  | Yes | 73 (100%) | 22 (0.9%) | 16 (1.3%) | 95 (6.8%) |  |
| Diabetes | No | 30 (41.1%) | 1297 (53.5%) | 1113 (91.3%) | 436 (31.4%) | <.001 |
|  | Yes | 43 (58.9%) | 1128 (46.5%) | 106 (8.7%) | 951 (68.6%) |  |
| Cancer | No | 50 (68.5%) | 2337 (96.4%) | 1034 (84.8%) | 1138 (82%) | <.001 |
|  | Yes | 23 (31.5%) | 88 (3.6%) | 185 (15.2%) | 249 (18%) |  |
| Glucose (mmol/L) | Median (IQR) | 6.3 (5.4 to 9.7) | 5.0 (4.7 to 5.4) | 5.4 (4.9 to 6.2) | 5.4 (4.9 to 6.2) | <.001 |
| HbA1c (mmol/mol) | Median (IQR) | 47.5 (38.8 to 63.9) | 34.4 (32.2 to 37.7) | 37.7 (34.4 to 42.1) | 37.7 (35.5 to 43.2) | <.001 |
