# Supplementary material for: Physical Activity Recommendations Tailored by a Predictive Model for Adults With High Blood Pressure: Observational Study
Source: J Med Internet Res. 2026 Jan 9;28:e78492. doi: 10.2196/78492 (PMC12788716; doi:10.2196/78492)
Supplement: Multimedia Appendix 12 [file jmir-v28-e78492-s012.docx]

**Multimedia Appendix 12.** The results of the sensitivity analyses


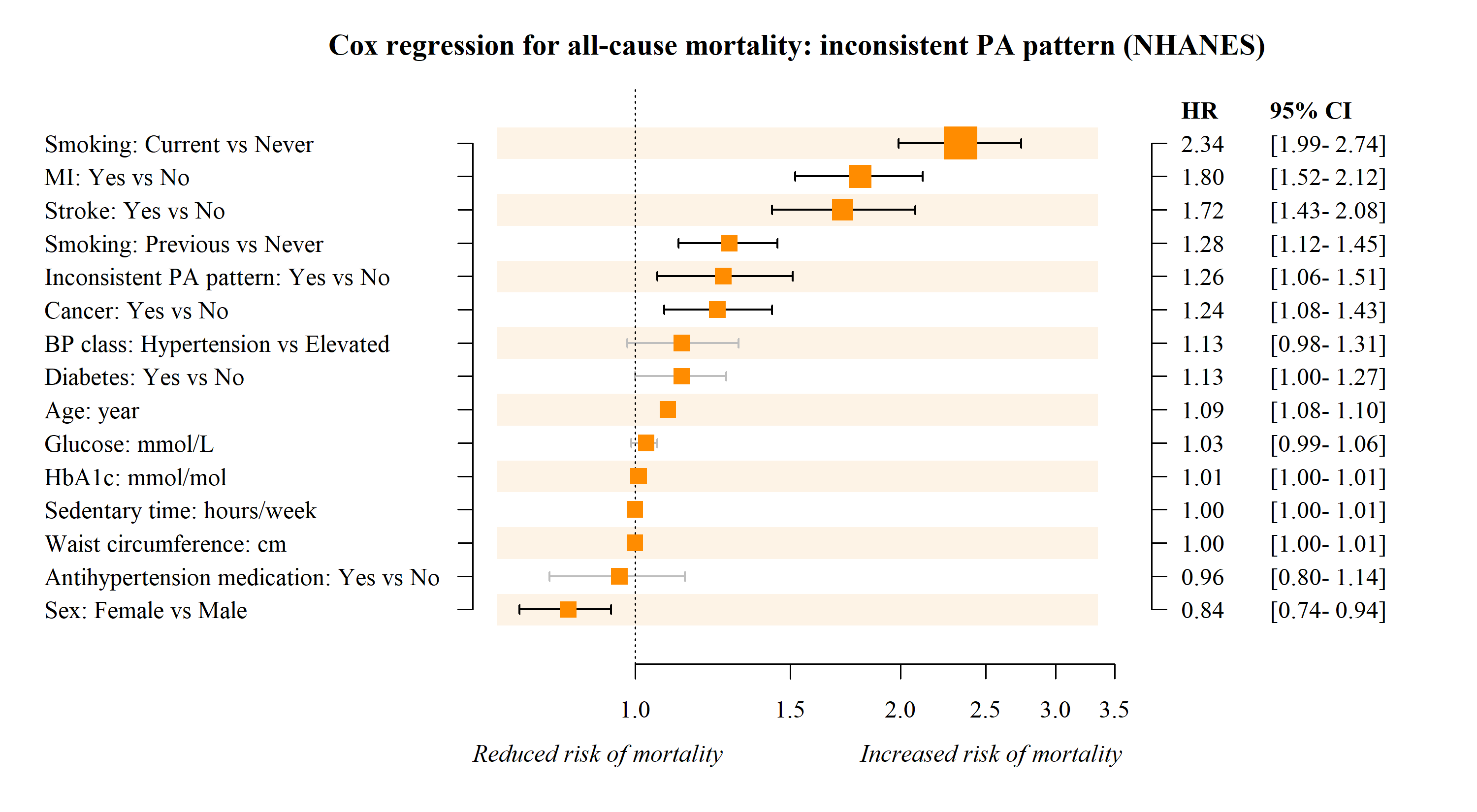


Figure 1. The association between inconsistent PA pattern and all-cause mortality in the NHANES cohort.


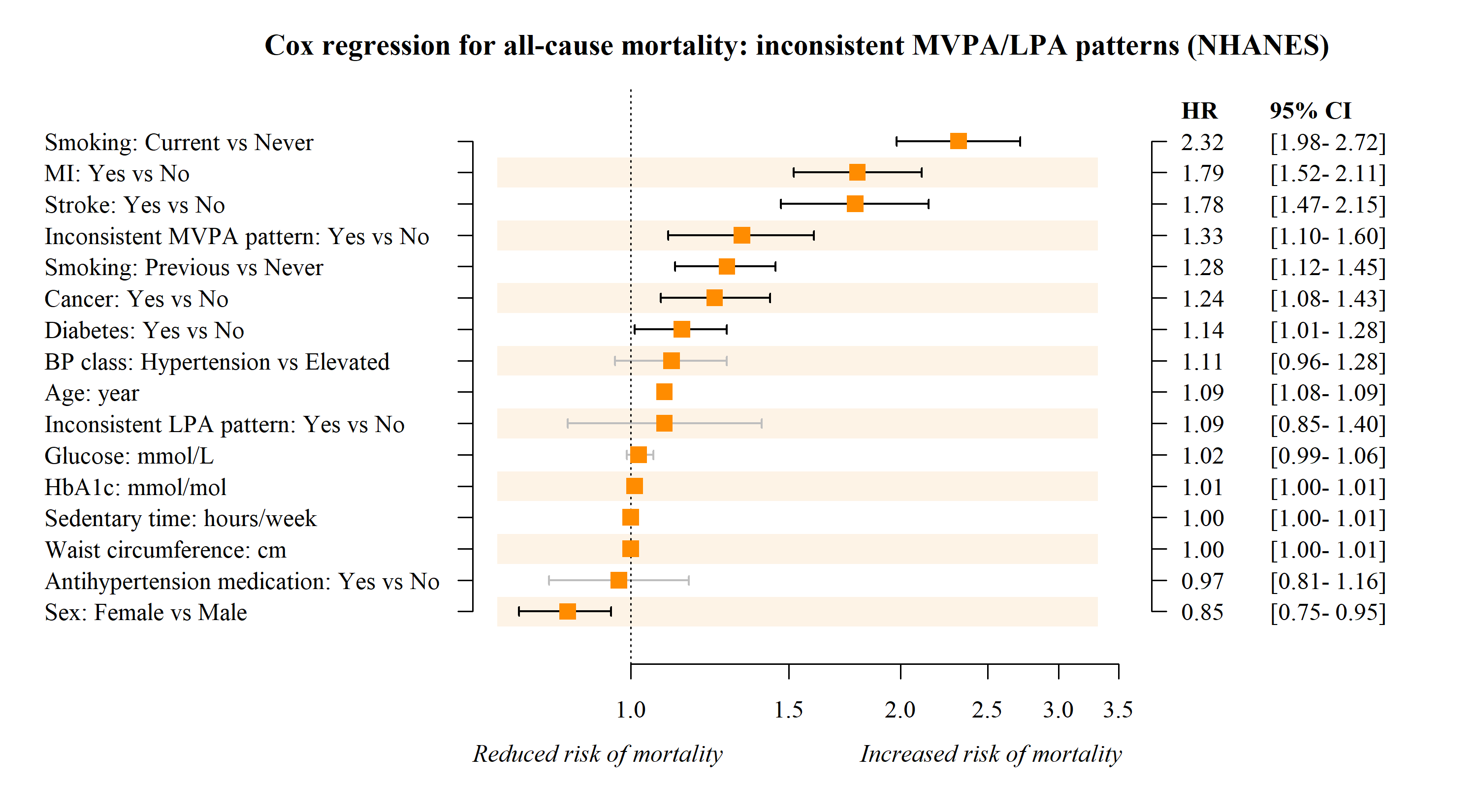


Figure 2. The association between inconsistent MVPA/LPA pattern and all-cause mortality in the NHANES cohort.


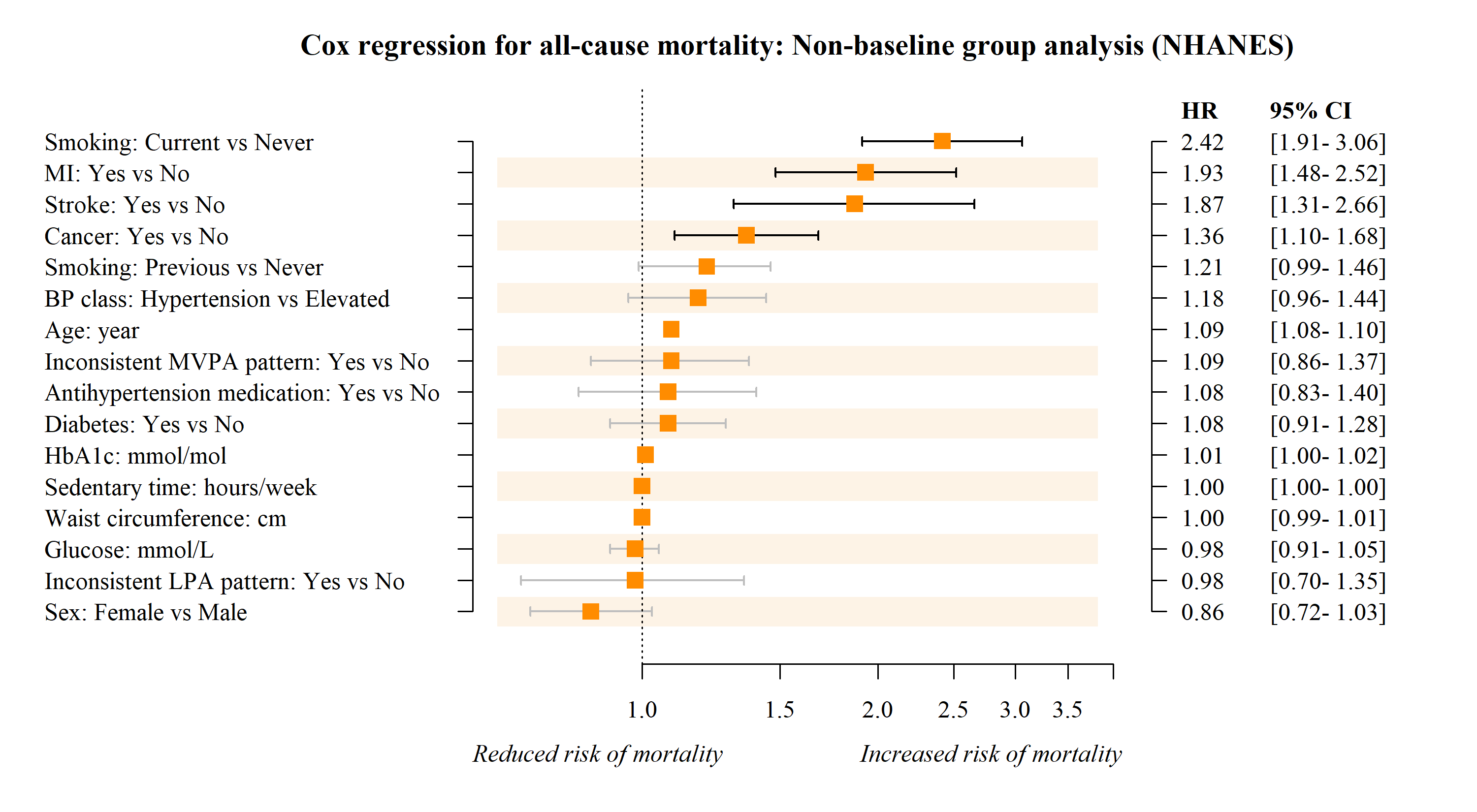


Figure 3. Subgroup analysis of the association between inconsistent MVPA/LPA pattern and all-cause mortality in the NHANES cohort.


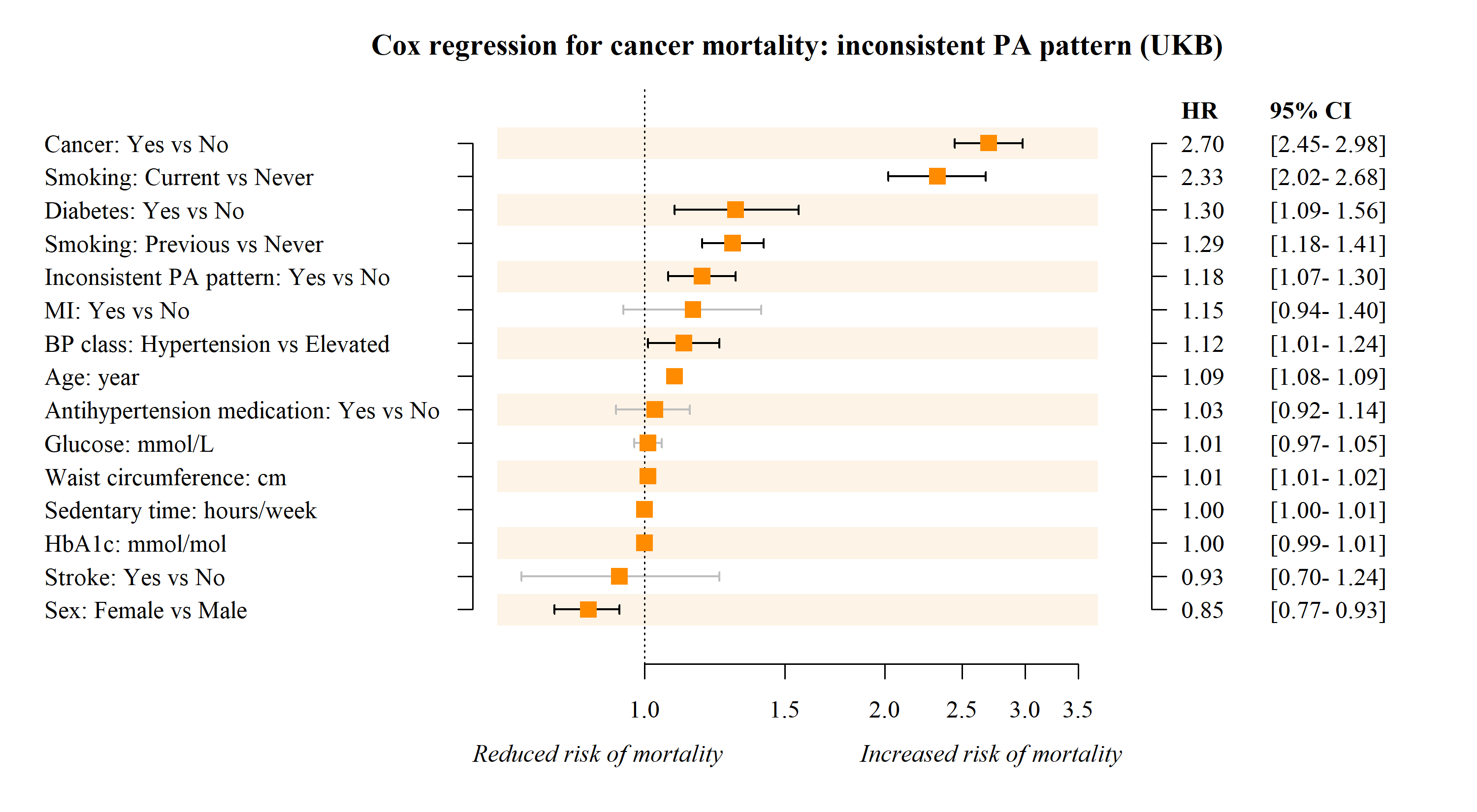


Figure 4. The association between inconsistent PA pattern and cancer mortality in the UKB cohort.


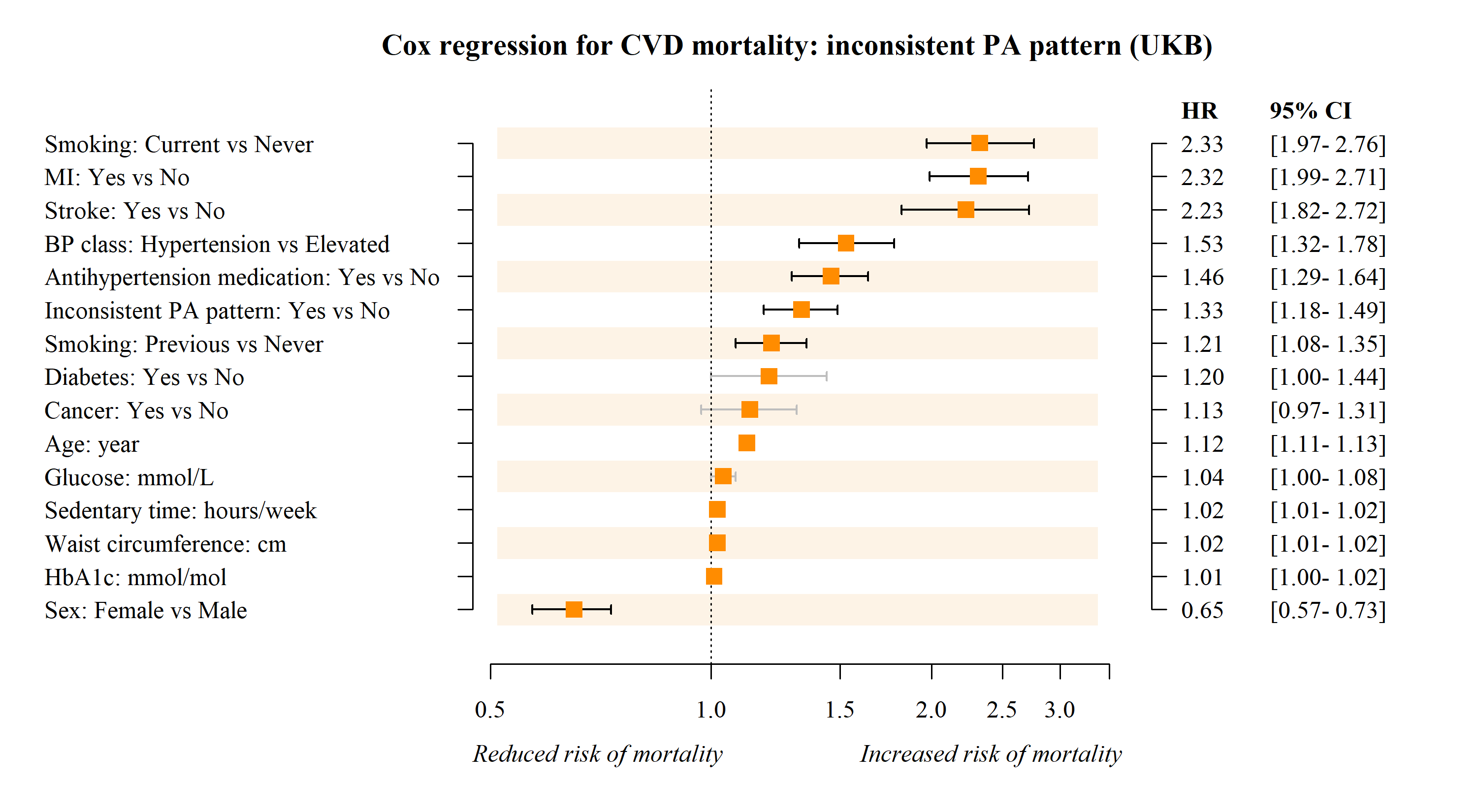


Figure 5. The association between inconsistent PA pattern and CVD mortality in the UKB cohort.


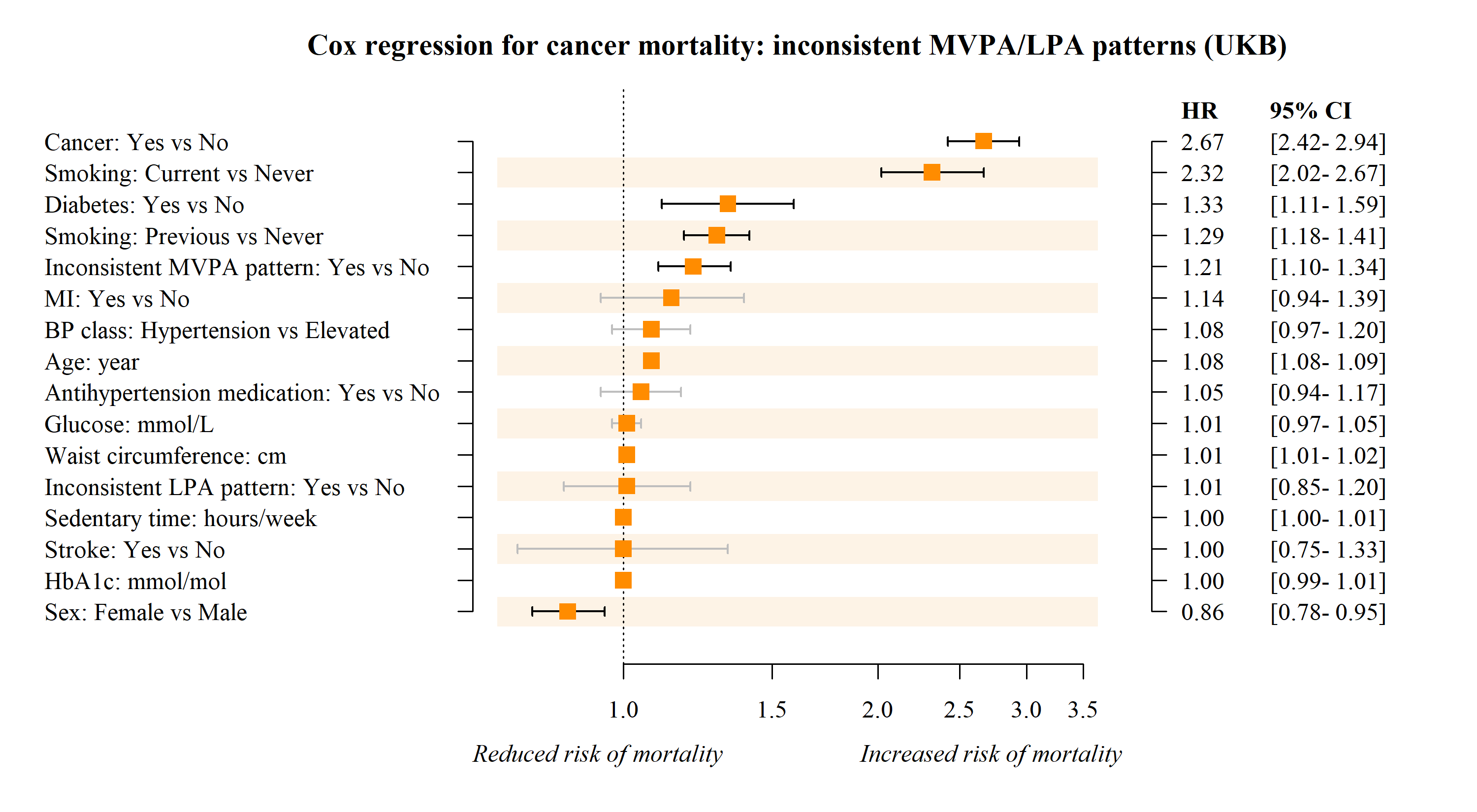


Figure 6. The association between inconsistent MVPA/LPA pattern and cancer mortality in the UKB cohort.


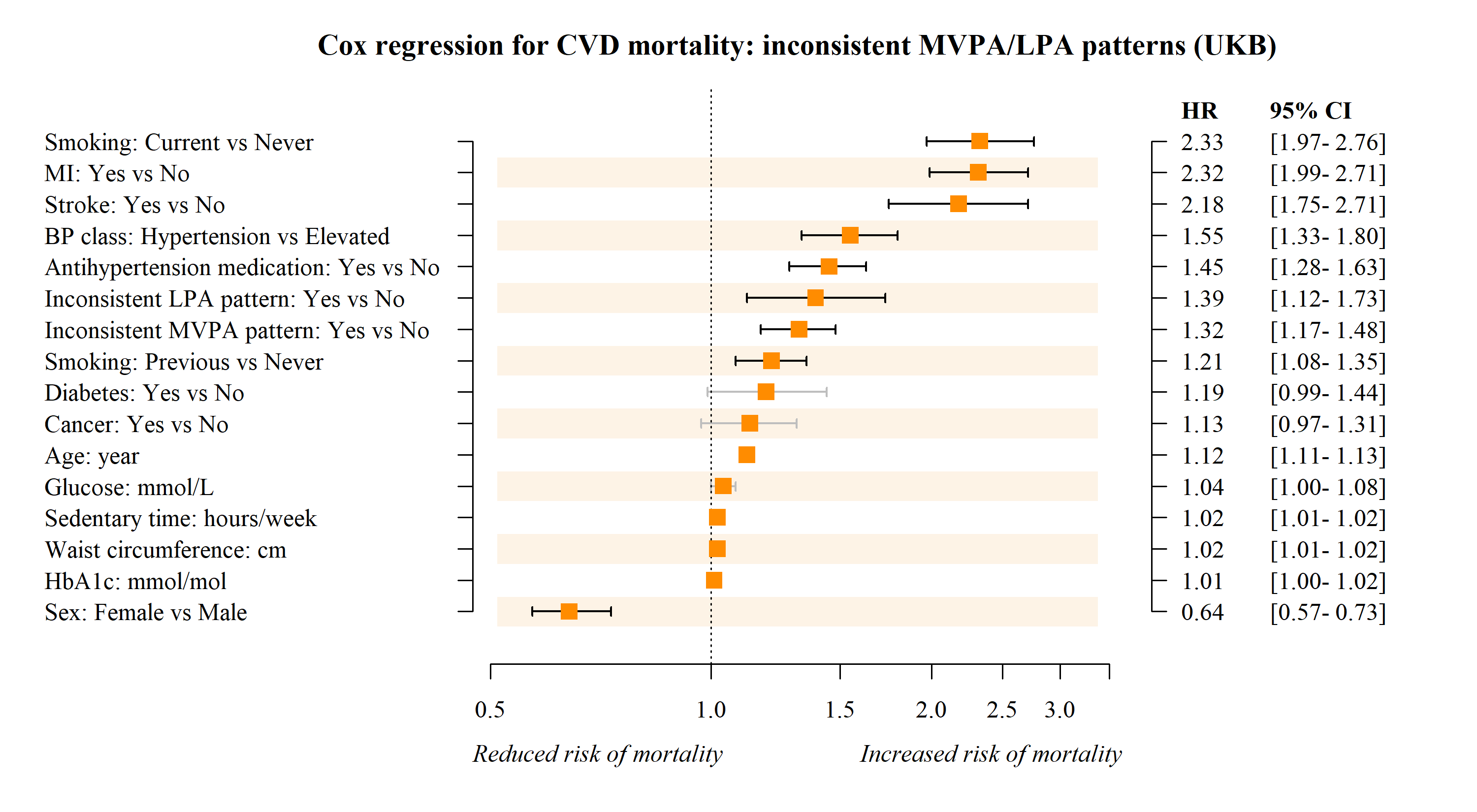


Figure 7. The association between inconsistent MVPA/LPA pattern and CVD mortality in the UKB cohort.


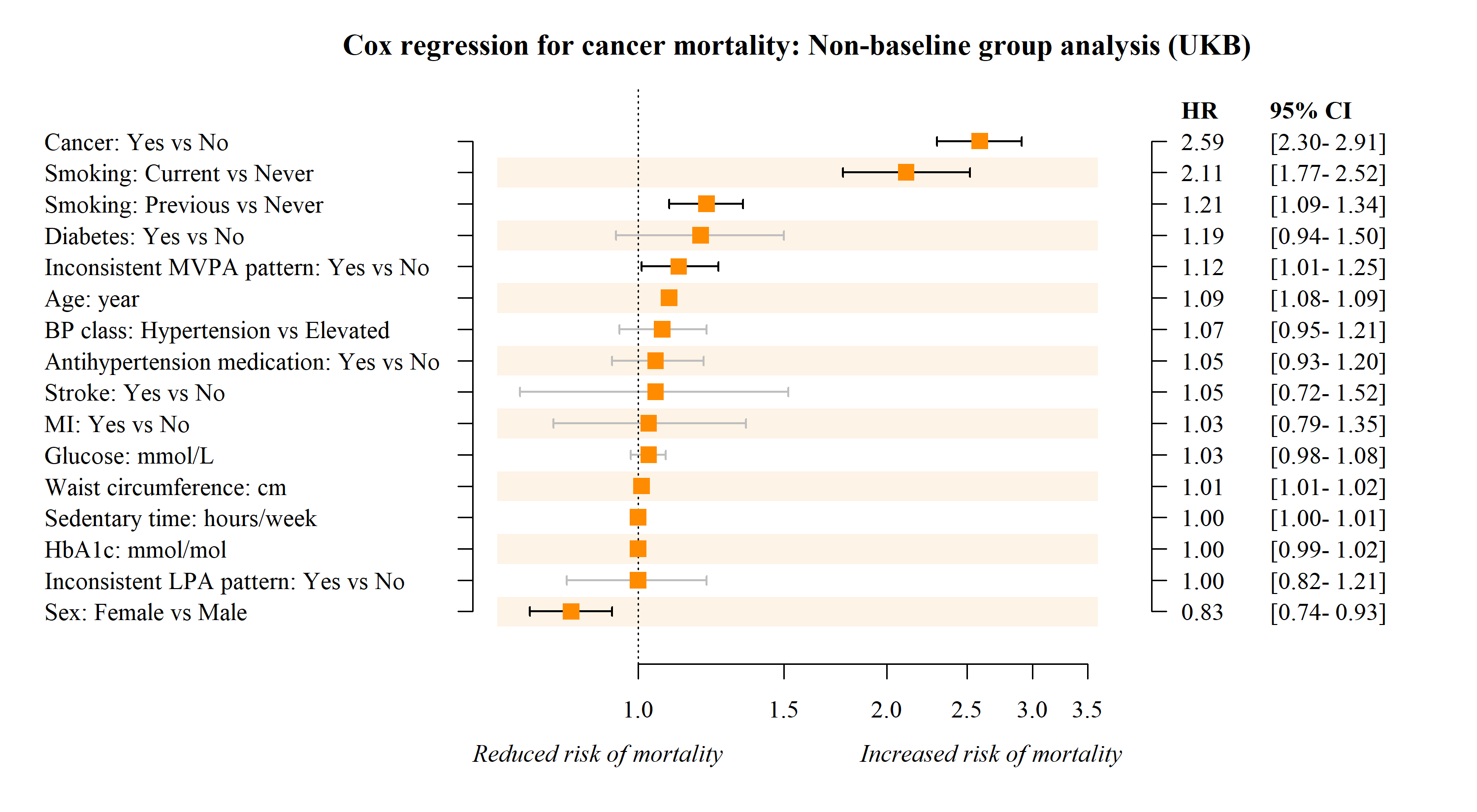


Figure 8. Subgroup analysis of the association between inconsistent MVPA/LPA pattern and cancer mortality in the UKB cohort.


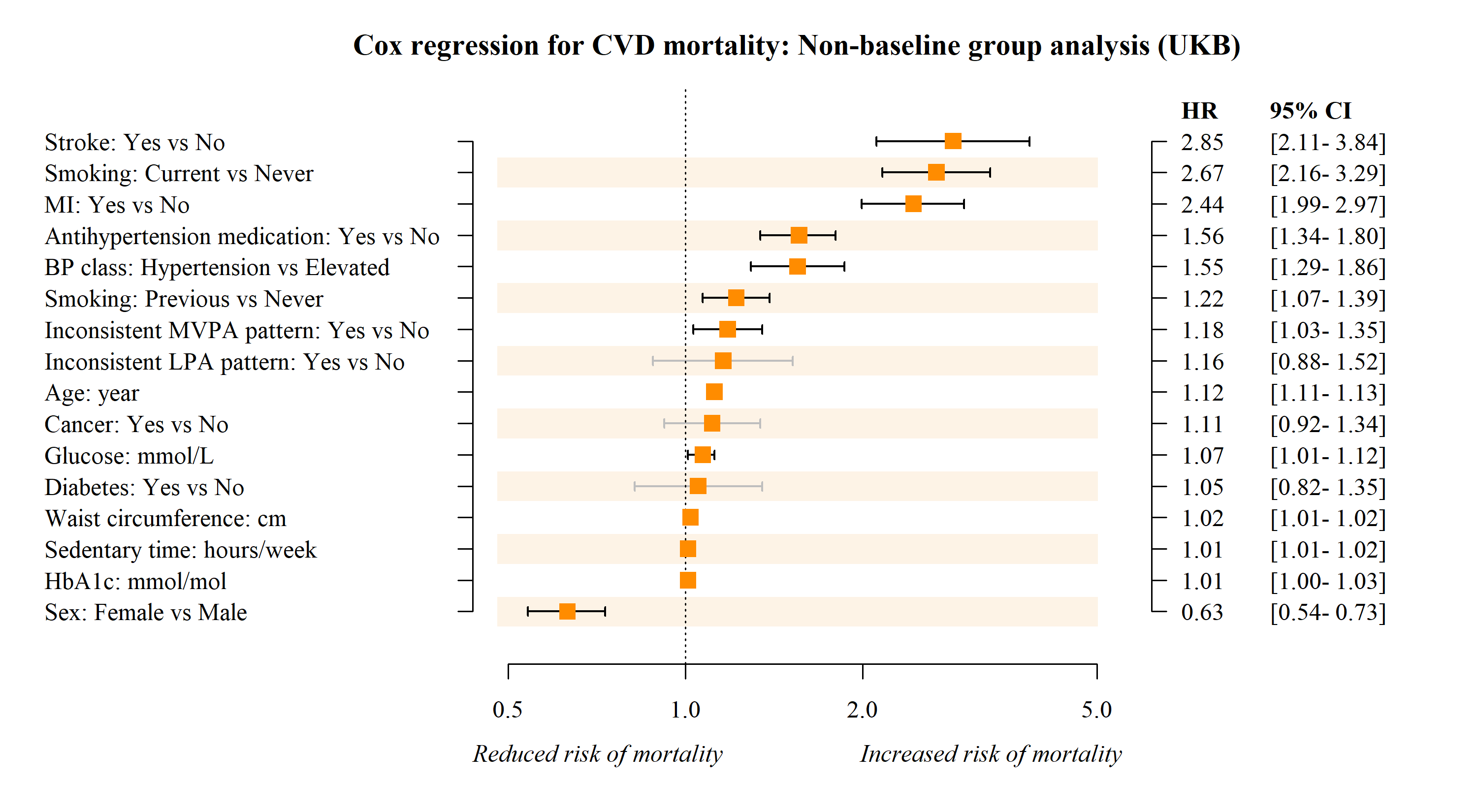


Figure 9. Subgroup analysis of the association between inconsistent MVPA/LPA pattern and CVD mortality in the UKB cohort.


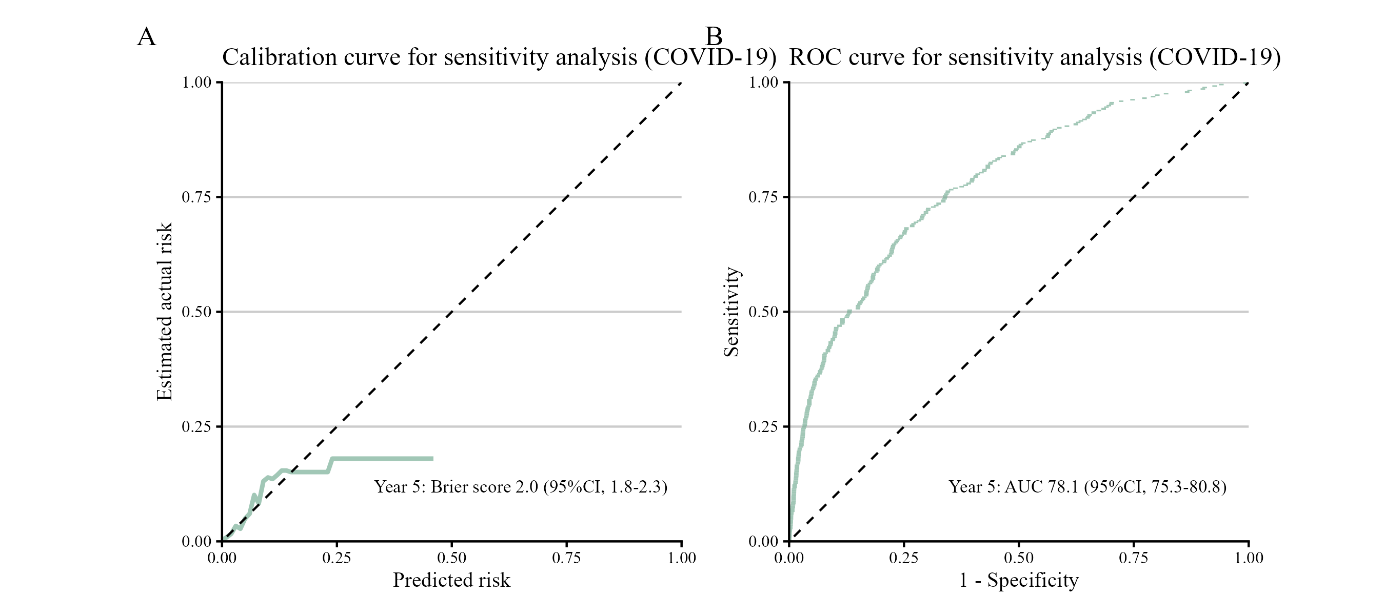


Figure 10. The model performance in pre-pandemic dataset for sensitivity analysis.


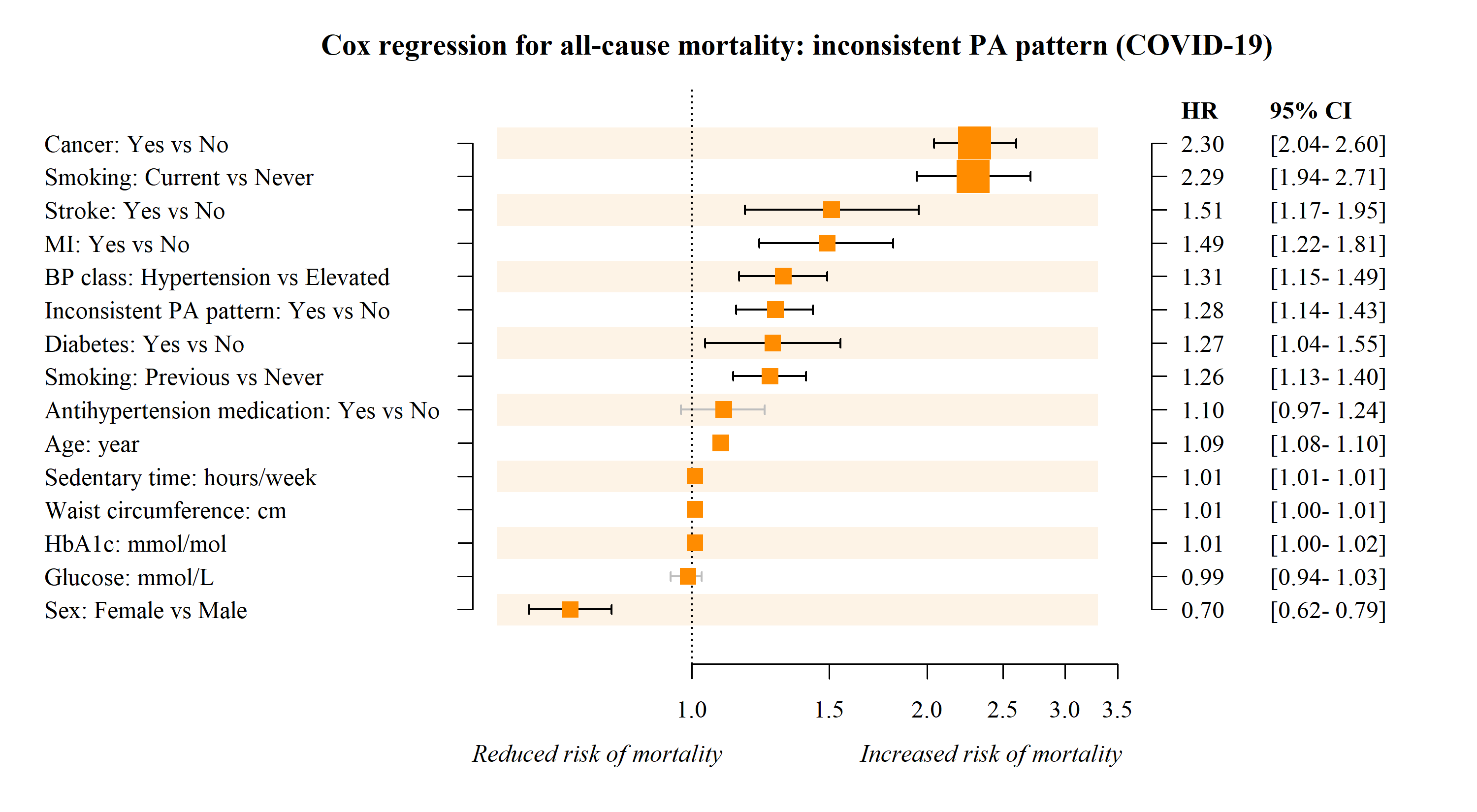


Figure 11. The association between inconsistent PA pattern and all-cause mortality in the pre-pandemic dataset.


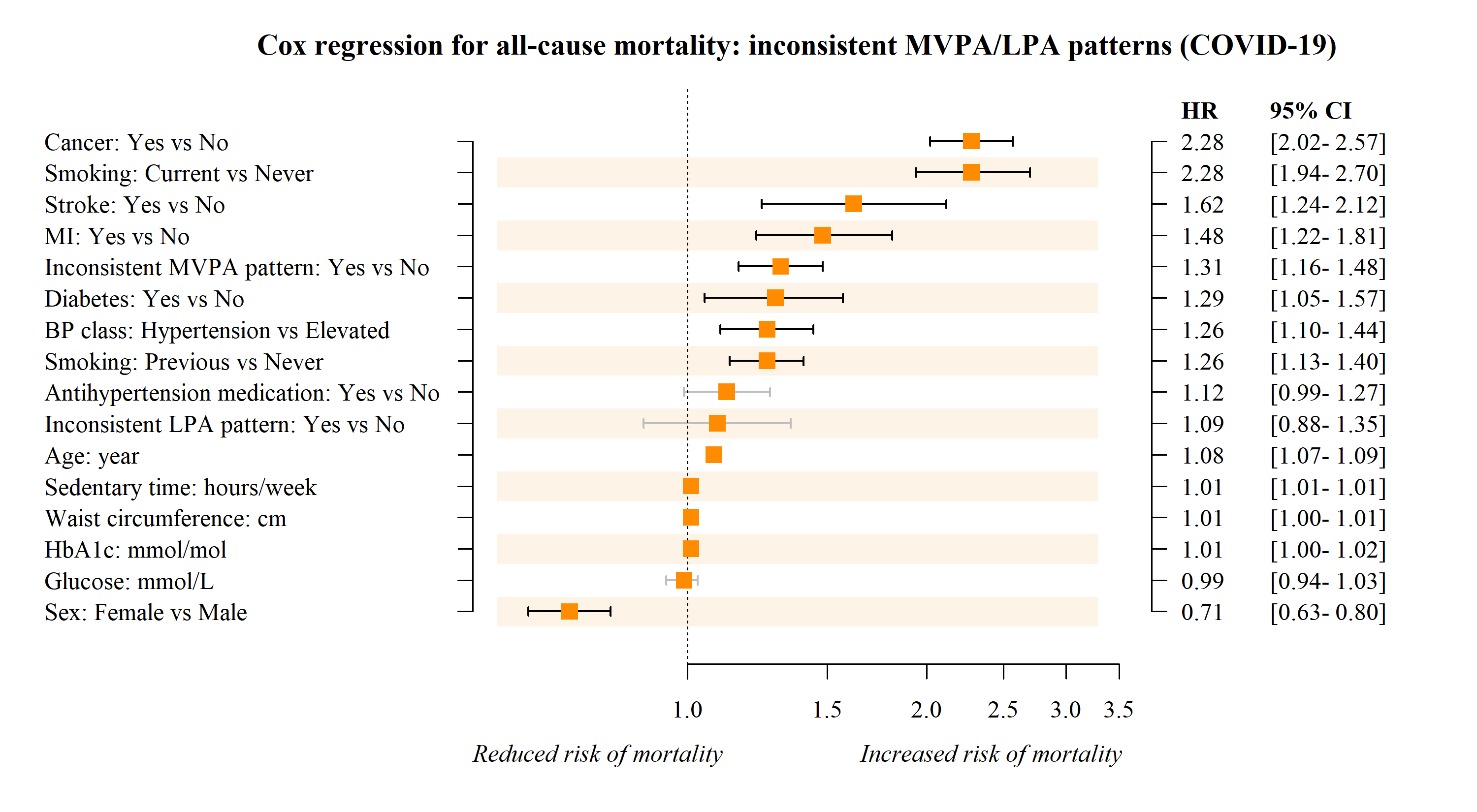


Figure 12. The association between inconsistent MVPA/LPA patterns and all-cause mortality in the pre-pandemic dataset.


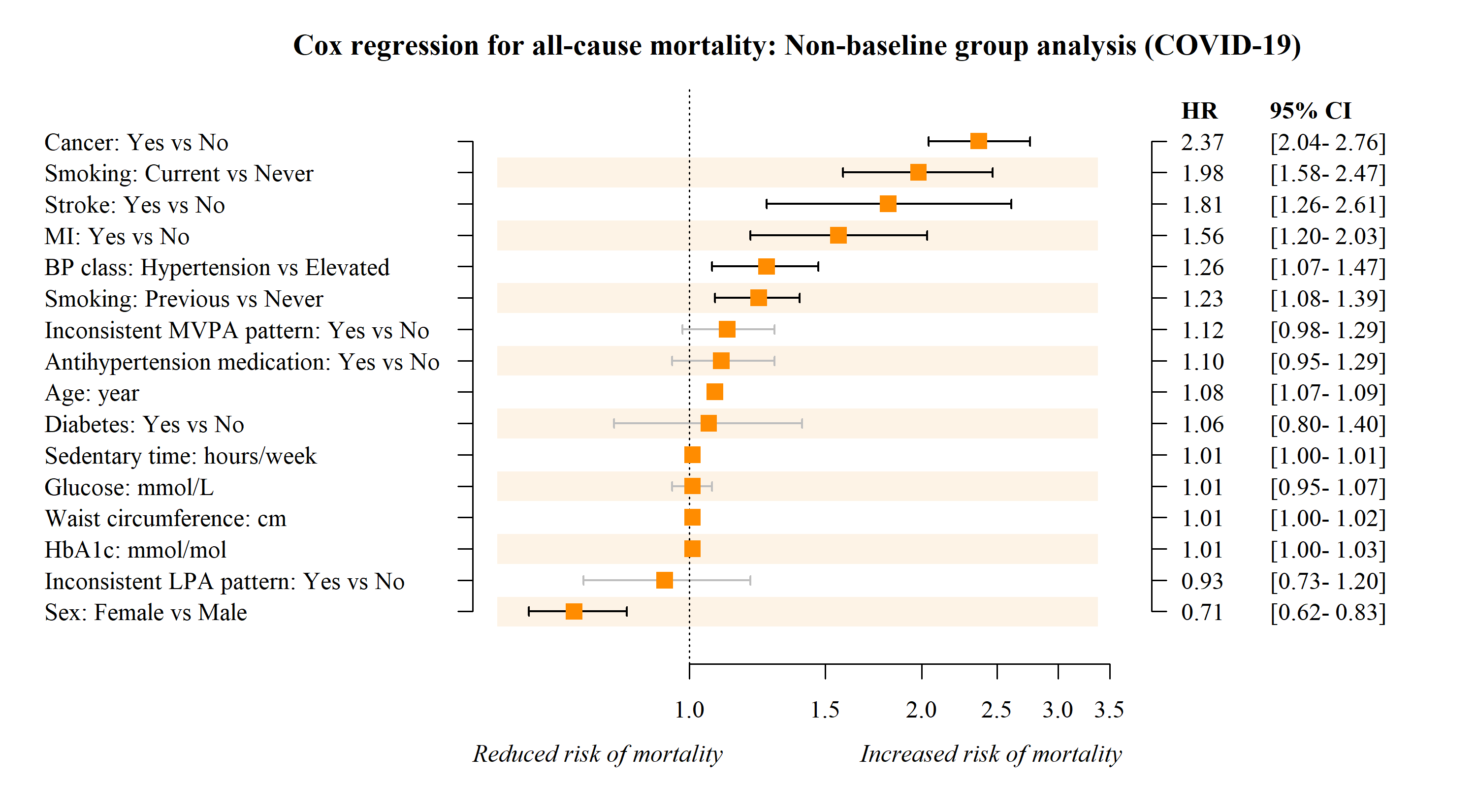


Figure 13. Subgroup analysis of the association between inconsistent MVPA/LPA pattern and all-cause mortality in the pre-pandemic dataset.


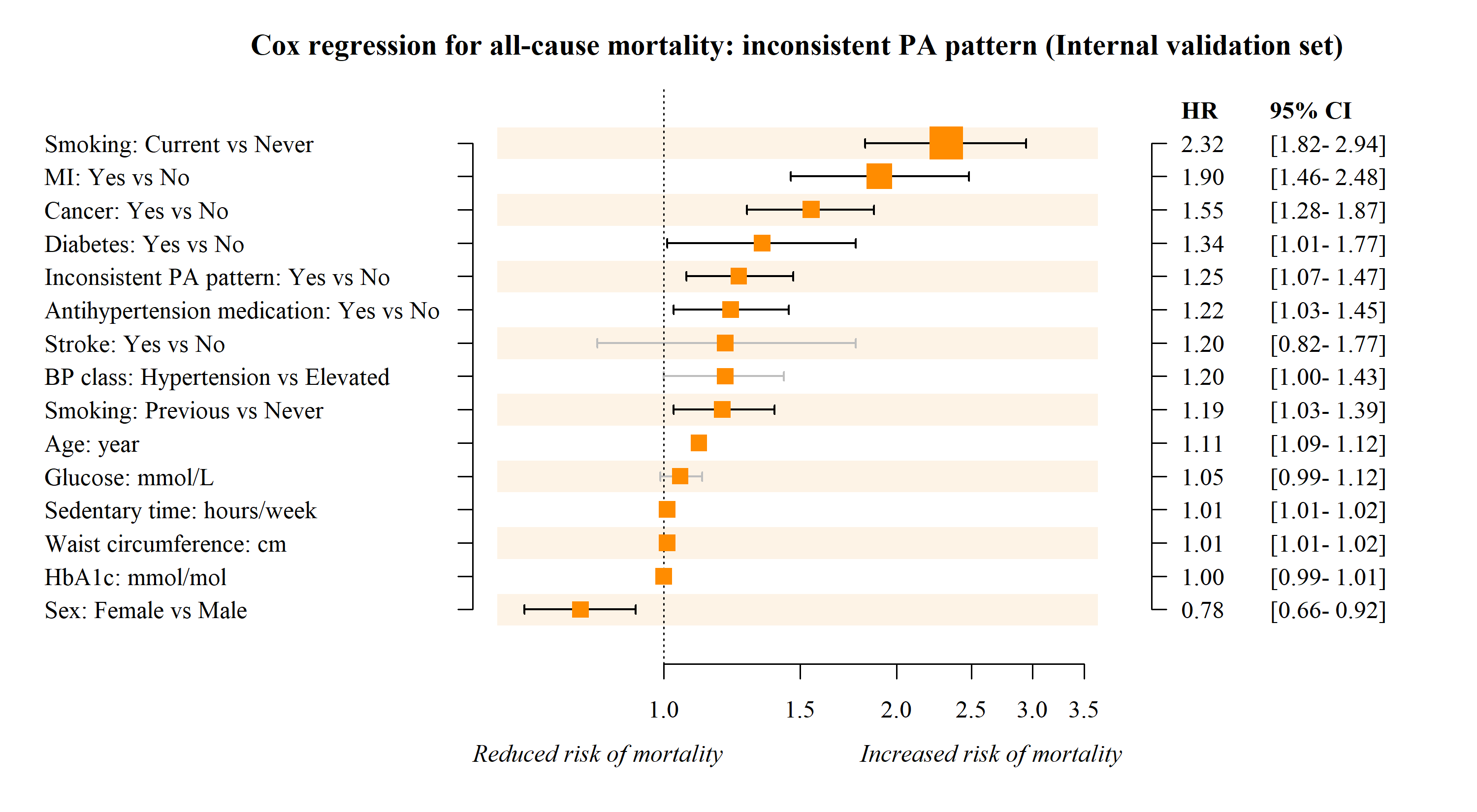


Figure 14. The association between inconsistent PA pattern and all-cause mortality in the internal validation set.


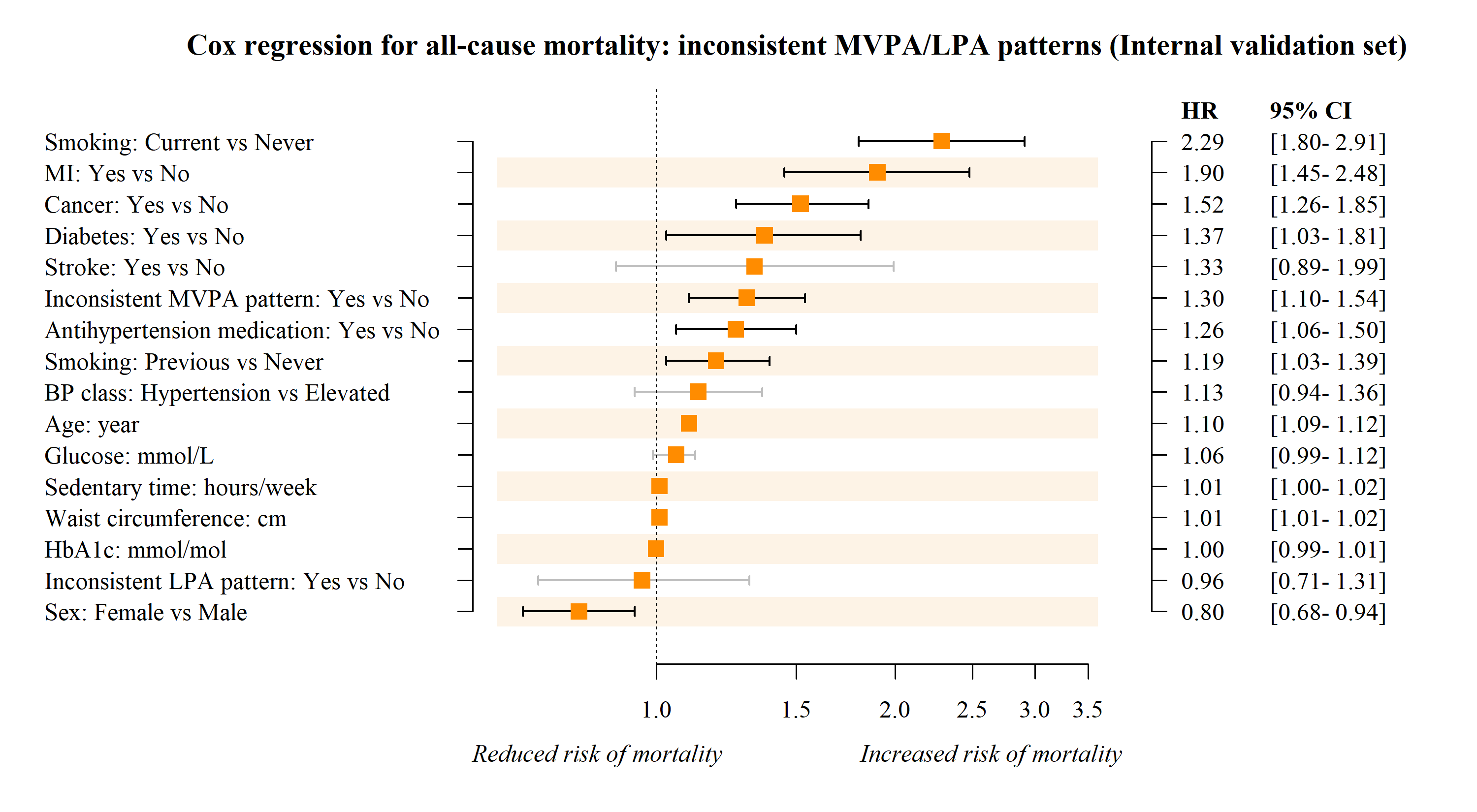


Figure 15. The association between inconsistent MVPA/LPA patterns and all-cause mortality in the internal validation set.


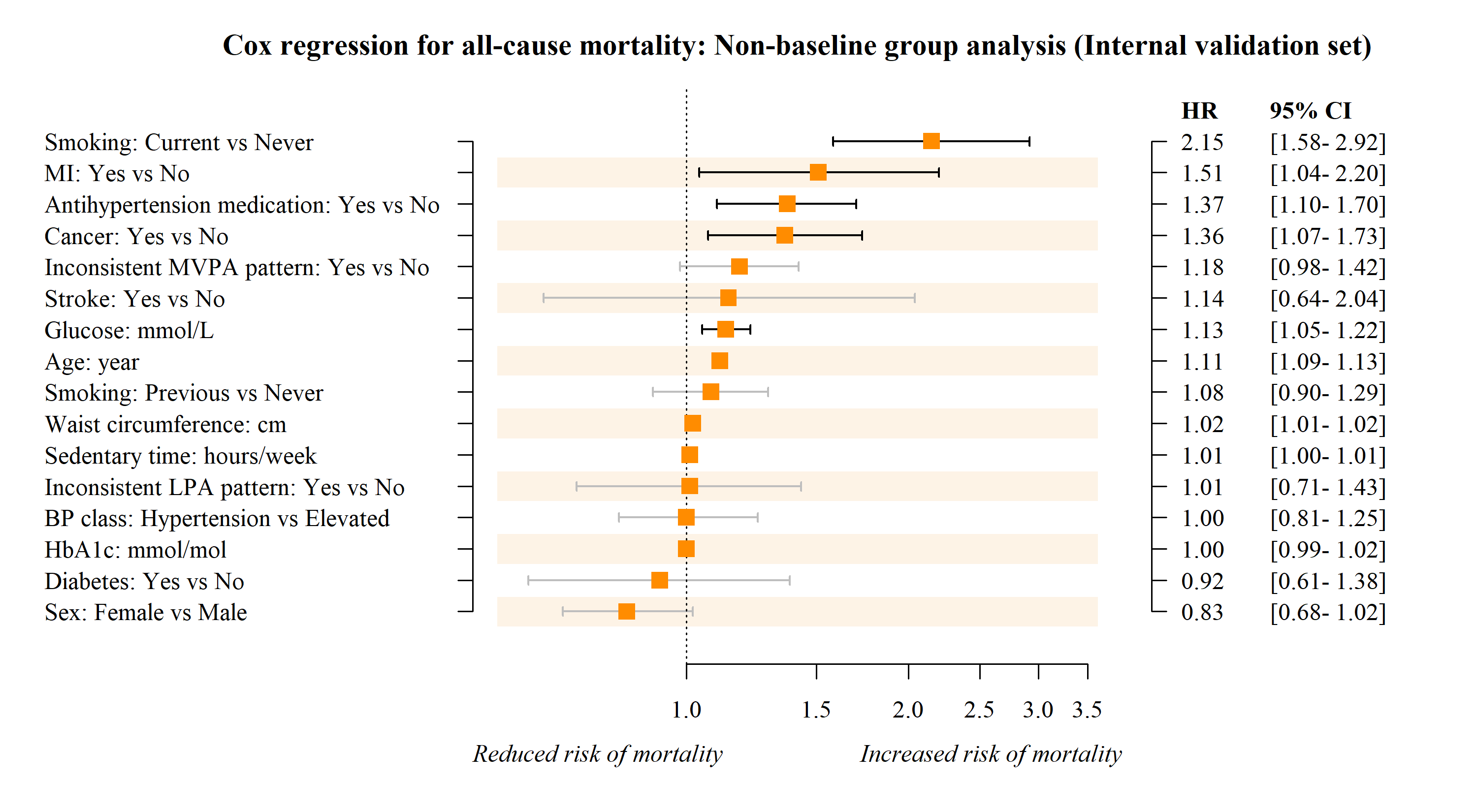


Figure 16. Subgroup analysis of the association between inconsistent MVPA/LPA pattern and all-cause mortality in the internal validation set.
